# Analyzing quality of life among people with opioid use disorder from the National Institute on Drug Abuse Data Share initiative: implications for decision making

**DOI:** 10.1007/s11136-024-03729-6

**Published:** 2024-08-08

**Authors:** Thomas Patton, Jan R. Boehnke, Ravi Goyal, Andrea Manca, Carla Marienfeld, Natasha K. Martin, Bohdan Nosyk, Annick Borquez

**Affiliations:** 1 Division of Infectious Diseases & Global Public Health, UC San Diego, 9500 Gilman Dr., La Jolla, San Diego, CA 92093 USA; 2 School of Health Sciences, University of Dundee, Nethergate, Dundee, DD1 4HN UK; 3 Centre for Health Economics, University of York, Heslington, York YO10 5DD UK; 4 UC San Diego Health Psychiatry, 8950 Villa La Jolla Drive, La Jolla, CA 92037 USA; 5 Faculty of Health Sciences, Simon Fraser University, Blusson Hall, Room 11300, 8888 University Drive, Burnaby, BC V5A 1S6 Canada

**Keywords:** Opioids, Cost-effectiveness, Withdrawal, Economics

## Abstract

**Purpose:**

We aimed to estimate health state utility values (HSUVs) for the key health states found in opioid use disorder (OUD) cost-effectiveness models in the published literature.

**Methods:**

Data obtained from six trials representing 1,777 individuals with OUD. We implemented mapping algorithms to harmonize data from different measures of quality of life (the SF-12 Versions 1 and 2 and the EQ-5D-3 L). We performed a regression analysis to quantify the relationship between HSUVs and the following variables: days of extra-medical opioid use in the past 30 days, injecting behaviors, treatment with medications for OUD, HIV status, and age. A secondary analysis explored the impact of opioid withdrawal symptoms.

**Results:**

There were statistically significant reductions in HSUVs associated with extra-medical opioid use (-0.002 (95% CI [-0.003,-0.0001]) to -0.003 (95% CI [-0.005,-0.002]) per additional day of heroin or other opiate use, respectively), drug injecting compared to not injecting (-0.043 (95% CI [-0.079,-0.006])), HIV-positive diagnosis compared to no diagnosis (-0.074 (95% CI [-0.143,-0.005])), and age (-0.001 per year (95% CI [-0.003,-0.0002])). Parameters associated with medications for OUD treatment were not statistically significant after controlling for extra-medical opioid use (0.0131 (95% CI [-0.0479,0.0769])), in line with prior studies. The secondary analysis revealed that withdrawal symptoms are a fundamental driver of HSUVs, with predictions of 0.817 (95% CI [0.768, 0.858]), 0.705 (95% CI [0.607, 0.786]), and 0.367 (95% CI [0.180, 0.575]) for moderate, severe, and worst level of symptoms, respectively.

**Conclusion:**

We observed HSUVs for OUD that were higher than those from previous studies that had been conducted without input from people living with the condition.

**Supplementary Information:**

The online version contains supplementary material available at 10.1007/s11136-024-03729-6.

## Abstract (plain language summary)

Thus far, health-related quality of life estimates for patients with opioid use disorder in the United States are limited, and importantly, they were not generated from studies among people living with the condition. This study extracted data from six clinical trials providing data among 1,777 people with opioid use disorder, made publicly available by the National Institutes of Health, to produce estimates of health-related quality of life. Our study found higher health-related quality of life estimates as compared to previous studies, modest impact of medications for opioid use disorder and strong impact of withdrawal symptoms on this outcome. These higher values among people with opioid use disorder might reflect the very negative perception of this condition among members of the general population (among whom these estimates have been generated previously). However, these relatively high estimates could also reflect an adaptation to the condition or a lack of awareness of associated-health damage in the context of dependence. The low number of observations providing data on medications for opioid use disorder led to high uncertainty around related estimates of health-related quality of life, but our findings could also reflect real experiences by patients in the absence of the positive effects of non-medication opioids, which deserve more attention in clinical practice. Our study suggests that systematically measuring withdrawal symptoms and representing these in health economic models might provide a more accurate representation of health-related quality of life among people with opioid use disorder and therefore of the impact and cost-effectiveness of interventions.

**Supplementary Information:**

The online version contains supplementary material available at 10.1007/s11136-024-03729-6.

## Clinical trial registration details

The information reported here results from secondary analyses of data from clinical trials conducted by the National Institute on Drug Abuse (NIDA). Specifically, data from NIDA-CTN-0001 (NCT00032955 and ’Buprenorphine/Naloxone versus Clonidine for Inpatient Opiate Detoxification’), NIDA-CTN-0002 (NCT00032968 and ‘Buprenorphine/Naloxone versus Clonidine for Outpatient Opiate Detoxification’), NIDA-CTN-0009 (NCT00067158 and ‘Smoking Cessation Treatment with Transdermal Nicotine Replacement Therapy in Substance Abuse Rehabilitation Programs’), NIDA-CTN-0030 (NCT00316277 and ‘A Two-Phase Randomized Controlled Clinical Trial of Buprenorphine/Naloxone Treatment Plus Individual Drug Counseling for Opioid Analgesic Dependence’), NIDA-CTN-0049 (NCT01612169 and ‘Project HOPE - Hospital Visit as Opportunity for Prevention and Engagement for HIV-Infected Drug Users’), NIDA-CTN-0051 (NCT02032433 and ‘Extended-Release Naltrexone vs. Buprenorphine for Opioid Treatment’) were included. NIDA databases and information are available at (https://datashare.nida.nih.gov). Additional observational data was used to equate different measures of health-related quality of life onto the same scale using psychometric methods. These data included the National Health Measurement Study (which is available at https://www.disc.wisc.edu/) and the Household Component files for the Medical Expenditure Panel Survey (which is available at https://meps.ahrq.gov/mepsweb/).

**Supplementary Information:**

The online version contains supplementary material available at 10.1007/s11136-024-03729-6.

## Introduction

Opioid use disorder (OUD) represents a significant public health challenge in the United States (US) as more than 700,000 people have died from an opioid overdose since 1999 [1]. Cost-effectiveness analysis of interventions to mitigate OUD-related health harms is an important tool for guiding public policy responses [2]. Cost-effectiveness studies typically rely on models that represent the condition’s clinical progression through different health states for which the health-related quality of life (HRQoL) has been previously estimated and expressed in terms of health state utility values (HSUVs) on a scale with reference points at 0 (dead) and 1 (perfect health) [3]. The process of estimating HSUVs, which are used to calculate quality-adjusted life years (QALYs) in cost-effectiveness models, is a challenging endeavor [4] and a recent study by Barbosa and colleagues observed that there is a dearth of evidence enabling their estimation for OUD [5]. When looking at the use of HSUVs in OUD models, Barbosa et al. showed that there was repeated use of one source of evidence across models in the published literature [6]. Despite the extensive use of these estimates, the values were elicited in a sample of British participants and so are not representative of the health preferences of the US, which is the focus of the current paper. HSUVs should ideally reflect the preferences of the jurisdiction under investigation given that there are important differences between values elicited in different countries [7]. More recently, a study by Wittenberg and colleagues elicited HSUVs in a US population-representative sample (*n* = 2,054) to determine their perception of the quality of life effects of OUD [8].

The primary objective of our study is to provide a new set of HSUVs for OUD in the US based on data collected in people experiencing the key health states of interest from multiple studies funded by the National Institute on Drug Abuse (NIDA) using an “off-the-shelf” value set associated with the descriptive system of the EQ-5D-3 L. While HRQoL data are routinely collected in NIDA-funded clinical trials [9], the instruments used vary across studies which undermines their comparability [10], potentially affecting cost-effectiveness assessments [11]. We resolved this issue by employing mapping algorithms to estimate HSUVs [12]. Having harmonized the heterogeneous HRQoL data, we estimated HSUVs for twelve OUD states, corresponding to different combinations of the following factors: opiate type, frequency and mode of administration, receipt of medications for OUD, and HIV diagnosis. The rationale for the selection of these factors was based on a review of cost-effectiveness models of interventions addressing OUD in the published literature, which found that they corresponded to the most frequently observed states with HSUVs assigned to them for the estimation of QALYs. To identify key drivers of HRQoL among people living with OUD, we investigated associations between these factors, as well as age, and HSUV estimates through conducting a series of regression analyses. A secondary objective of the paper is to explore the impact of the withdrawal symptoms associated with OUD when estimating HSUVs for OUD cost-effectiveness models. Despite being a fundamental concern for patients with OUD, no existing models have sought to reflect these symptoms, raising a concern about the representation of the experiences of people with OUD in cost-effectiveness models [13–15].

## Methods

### Evidence of requirements for economic models in OUD

Following recommended methods [4], we established which health states are most important in cost-effectiveness models for OUD. Existing cost-effectiveness models in OUD were examined to identify the defined health states that had HSUVs assigned to them for the estimation of QALYs. Existing studies were selected from systematic reviews of cost-effectiveness studies in OUD [5, 16, 17]. Cost-effectiveness studies released after the publication of the systematic reviews were also obtained by searching studies citing the reviews on Google Scholar. Table 1 presents a list of variables related to the health states represented in the models identified. The variable most frequently represented was reported (or suspected) extra-medical opioid use, followed by treatment with medications for OUD. Other key descriptors include drug-injecting behaviors, HIV infection status, and hepatitis C virus infection status.

**Table 1:** Variables associated with health state utility values in opioid use disorder (OUD) cost-effectiveness models

Variable	References
Reported or suspected extra-medical opioid use (i.e. model assigns different values to patients depending on their levels of opioid use outside of treatment)	[5, 6, 18–32]
Reported type of extra-medical opioid use (i.e. the model assigns different values depending on whether patients use prescription opioids or heroin outside of treatment)	[19]
Reported injection as mode of administration for opioid use (i.e. the model assigns different values depending on whether patients inject opioids)	[5, 23, 27, 31, 33]
Receipt of medications for treatment of OUD (i.e. the model assigns different values depending on whether patients receive medicated treatments for OUD)	[5, 18, 20–26]
HIV infection status (i.e. the model assigns different values to patients depending on their HIV status and disease stage)	[18, 20, 21, 23, 25, 31–33]
Hepatitis C virus infection status (i.e. the model assigns different values to patients depending on their hepatitis C virus status and disease stage)	[23, 34]
Incarceration (i.e. the model assigns different values depending on whether patients are incarcerated)	[18]
Pregnancy and birth outcomes (i.e. the model assigns different values depending on the outcomes experienced by pregnant women with OUD)	[26]
Hypoxia (i.e. the model captures the health state utility impact of patients experiencing hypoxia, which occurs when there is insufficient cerebral oxygenation following an overdose)	[30]

### Data obtained from the NIDA data share initiative

Trial data were acquired from the NIDA website if they fulfilled a series of inclusion criteria. First, they needed to have data collected in patients receiving any of the following diagnoses: (i) opioid dependence according to the definition set out in the fourth edition of the Diagnostic and Statistical Manual of Mental Disorders [35], (ii) OUD according to the definition set out in the fifth edition of the Diagnostic and Statistical Manual of Mental Disorders [36], (iii) opioids as the substance of major drug use as determined by an interviewer, or (iv) receiving methadone for OUD. Second, they needed generic measures of HRQoL collected in these patient populations, which yielded data collected using the EQ-5D-3 L, the 12-Item Short Form Survey (SF-12) Version 1 and the SF-12 Version 2 measures. Additional searches were conducted to identify alternative HRQoL measures with a view to expanding the number of trials that could be included in the analysis. Alternative measures listed in the NIDA website were cross-referenced against a list of all existing mapping algorithms [37] but no measures were identified that could be linked to preference-based instruments via mapping algorithms. Finally, the trials needed to collect variables aligning with the primary health states of interest in OUD models, namely those relating to the receipt of medications for OUD and reported extra-medical opioid use. There were no variables that made a distinction between patients being either engaged in extra-medical opioid use or not. Instead, a continuous variable was chosen capturing the number of days of extra-medical opioid use in the past 30 days. For the latter, extra-medical opioid use was captured using self-reported responses to the Addiction Severity Index (ASI) assessment tool, in addition to data from urine drug screening tests. Finally, data on self-reported withdrawal symptoms, as measured by the Subjective Opiate Withdrawal Scale (SOWS), was obtained for study NCT02032433 to explore the consistency of the regression results when this was included as a covariate. For each of the trials obtained, the associated documentation was checked to identify variables in Table 1. **Missing data**.

A description of missing data in the studies can be found in Appendix A. For observations with partially missing HRQoL data, the mapping procedure, described in the next section, was used to predict EQ-5D-3 L scores based on the items where data were not available.

### Methods for equating different health-related quality of life measures with external evidence

**Fig. 1:**
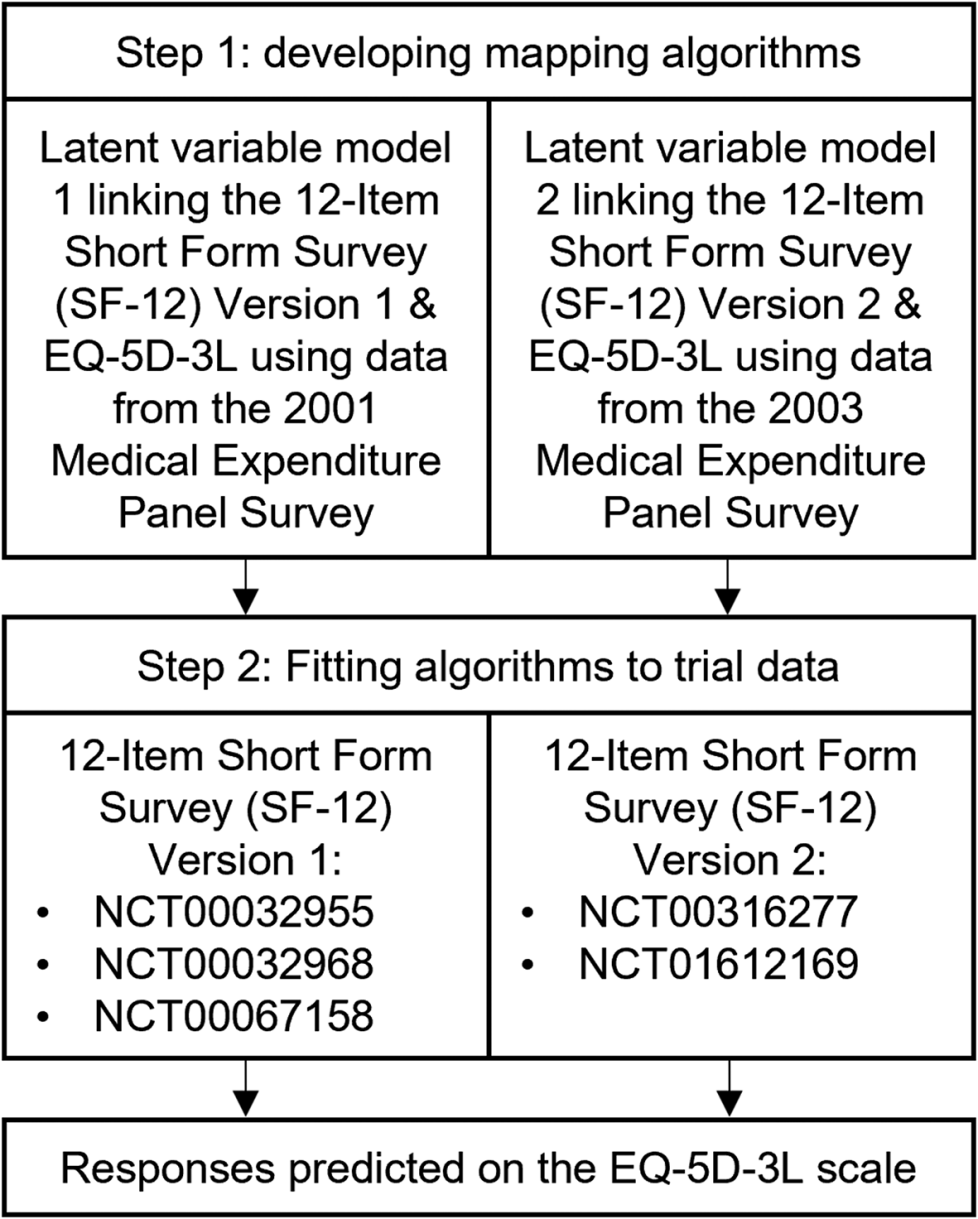
Flow diagram showing two-step mapping procedure for equating health-related quality of life measures

The EQ-5D-3 L was designated as the target measure for valuing HRQoL in this study for both evidential and practical reasons. First, previous research has validated its use in people with OUD through assessments of its content validity, construct validity, tests for evidence of floor and ceiling effects, and responsiveness [38]. Moreover, datasets were available that permitted the development of mapping algorithms between the EQ-5D-3 L and SF-12 measures. US population-based preference weights were assigned to EQ-5D-3 L responses to estimate health index values [39]. For studies that did not collect the EQ-5D-3 L, a two-step mapping procedure, shown in Fig. 1, was employed to predict HRQoL responses on the EQ-5D-3 L scale. The first step involved developing mapping algorithms to quantify the relationship between each version of the SF-12 and the EQ-5D-3 L. No studies were identified in the published literature mapping between the SF-12 Version 2 and the EQ-5D-3 L with US population values. Although previous studies have been conducted which map between the SF-12 Version 1 and the EQ-5D-3 L [40, 41], new mapping algorithms were developed to make use of recent methodological advances from the published literature. Methodological insights from the field of psychometrics research were integrated within a latent variable modeling framework to characterize the relationship between HRQoL measures in terms of a shared, latent factor, such that the different measures were assumed to capture alternative realizations of the same underlying health state [42, 43]. Separate mapping algorithms were required for the different versions of the SF-12 given that there are important differences in the item wording and response options [44, 45]. Two datasets from the Medical Expenditure Panel Survey [46, 47], collected in samples of the US general population, were obtained to equate different measures of HRQoL using a latent variable modeling framework. In accordance with the ‘Preferred Reporting Items for Studies Mapping onto Preference-based Outcome Measures: The MAPS Statement’ [48], details about the development of the mapping algorithms can be found in Appendices B and C in the supplementary materials.

### Statistical analysis of health state utility data

A regression analysis was conducted by fitting a mixed-effects model to the pooled HSUV data using the glmmTMB package in R [49]. A beta-binomial distribution was assumed to account for the bounded nature of HSUVs [50]. Random effects were applied to the model intercepts to account for correlations between repeated observations on the same study participant at different points in time.

The main fixed effects in the model included three variables on the self-reported number of days of opioid use in the past 30 days, corresponding to the use of heroin, other opiates and methadone respectively. A dummy variable was also included to indicate whether participants reported injecting as their main route of opioid administration. Other main fixed effects included age, HIV diagnosis, and a dummy variable indicating whether participants were receiving ongoing treatment with medications for OUD, specifically either methadone or buprenorphine. Naltrexone was not considered due to its fundamentally different treatment mechanism as an opioid antagonist. We sought to distinguish between the two variables corresponding to methadone use. One refers to methadone use as recorded either in the treatment log or in the concomitant medication records. The second variable corresponds to the self-reported number of days of methadone use in the past 30 days in the Addiction Severity Index questionnaire. Exploratory analyses indicate that responses to the latter variable overwhelmingly refer to methadone use outside of medicated settings (see Table D3 in Appendix D).

Interactions between the main fixed effects variables corresponding to opioid use (medicated or non-medicated) were included to account for participants using multiple types of opioids. This is a critical issue given that individuals’ drug choices are influenced by the availability of drug types, as well as financial and social considerations [51]. As such, it is reasonable to expect that the relationship between each of these variables and HSUVs might vary according to the remaining drug use variables in the regression model. All predictor variables were retained in the model regardless of whether they were significant or not because the exclusion of predictors from cost-effectiveness models on the grounds of statistical significance could bias the results if these predictors have important impacts on costs and health consequences [52, 53]. The inclusion of non-significant predictors in the model ensures that uncertainty can be appropriately characterized in cost-effectiveness assessments for decision making, including the value of further data collection to resolve sample uncertainty [3]. A sensitivity analysis was conducted to explore differences in outcomes across the studies (see Appendix F).

A secondary analysis was conducted in a subset of the data (study NCT02032433) that included the SOWS measure as an additional covariate to quantify the detrimental health effects of withdrawal symptoms. One hundred and forty-one observations were dropped from the NCT02032433 data due to missing SOWS data. A regression analysis was then run on this data. Only one of the study participants in the NCT02032433 data had an HIV positive diagnosis so this variable was dropped from the analysis. Aside from this change and the additional inclusion of the SOWS variable, the model specification was the same as that in the main analysis.

The R script for all the analyses in this study can be found in the supplementary materials. Our interpretations of the regression analyses are concerned with three sets of outputs: (i) the direct outputs from the regression analysis which show parameter estimates on the beta-binomial scale, (ii) contrasts of the estimated marginal effects for predictor variables to provide a more tangible interpretation of the results [54, 55], and (iii) adjusted predictions of HSUVs for different health states of interest in OUD models.

### Ethics approval

The Institutional Review Board of the University of California San Diego determined that an ethics review was not required because this study relied on the use of secondary de-identified data (Project #805,662).

## Results

### Datasets

Six studies meeting the inclusion criteria were identified through the NIDA Data Share Initiative. Table 2 provides information on the characteristics of these studies. These studies included data for five out of the nine variables listed in Table 1. In addition to the variables relating to the levels and types of extra-medical opioid use, the ASI captured information indicating whether participants reported injecting as their main route of opioid administration. One study (NCT01612169) captured data in people living with HIV and another (NCT02032433) collected information on participants’ HIV status at baseline. For the remaining datasets, study participants were assumed to be HIV negative. Two studies collected information on the presence of hepatitis C virus antibodies in study participants’ blood samples. Unfortunately, these data did not distinguish between active and resolved hepatitis C virus infection, so it was not possible to explore associations between HSUVs and the presence of active hepatitis C virus infection. In addition, data were not available to explore associations with either incarceration status, pregnancy, birth outcomes, or hypoxia. Table A1 in the supplementary materials describes the inclusion and exclusion of observations for the analysis. Just over 6% of observations were dropped, either due to a misalignment in the timing of the collection of variables (2.3%), a failure to provide any HRQoL responses (2.0%), missing responses for variables pertaining to extra-medical opioid use (1.1%), or because self-reported extra-medical opioid use conflicted with urine drug screen results (0.8%). The SOWS measure was collected in 91% observations in the NCT02032433 study.

**Table 2:** Characteristics of studies obtained from the NIDA Data Share Initiative

Clinical trial identifier	NCT00032955	NCT00032968	NCT00067158	NCT00316277	NCT01612169	NCT02032433
NIDA Clinical Trials Network number	CTN0001	CTN0002	CTN0009	CTN0030	CTN0049	CTN0051
Study period	2001–2002	2001–2002	2003–2004	2006–2009	2012–2015	2014–2017
Patient population	Opiate dependence diagnosis with DSM-IV criteria [35]	Opiate dependence diagnosis with DSM-IV criteria [35]	Subgroup with opiate dependence diagnosed with DSM-IV criteria [35] administered by a research assistant or study clinician.	Opiate dependence diagnosis with DSM-IV criteria [35]	Hospitalized HIV-infected PWUD – subgroup of individuals either with opioid use that was reported to be a major problem or receiving methadone for the treatment of OUD	Opioid-use disorder diagnosis with DSM-V criteria [36]
Interventions	BUP/NLX versus clonidine for medically supervised withdrawal	BUP/NLX versus clonidine for medically supervised withdrawal	Smoking cessation treatment as an adjunct to standard substance use treatment versus standard substance use treatment alone	*Phase 1*: four-week BUP/NLX treatment with taper, plus random assignment to SMM or EMM *Phase 2*: 12-week outpatient stabilization treatment with BUP/NLX, plus random assignment to SMM or EMM	Comparison of three interventions achieve HIV virologic suppression: Patient Navigator intervention versus Patient Navigator plus Contingency Management intervention versus Treatment as Usual	Extended-Release Naltrexone versus BUP/NLX
Setting	Inpatient	Outpatient	Outpatient	Outpatient	Hospital	Outpatient
HRQoL instrument	SF-12 Version 1 (Baseline, 1, 3 and 6 months)	SF-12 Version 1 (Baseline, 1, 3 and 6 months)	SF-12 Version 1 (Baseline, 8 weeks, 13 weeks, and 26 weeks)	SF-12 Version 2 (Baseline, final visit of phase 1, and 24 weeks in phase 2)	SF-12 Version 2 (Baseline, 6 months, and 12 months)	EQ-5D (Baseline, either 24 weeks or the end of treatment, and 36 weeks)
HIV diagnosis	None	None	None	None	All patients diagnosed as HIV-infective. Viral load and CD4 count collected at visits (Baseline, 6 months, and 12 months)	Only collected during the screening phase when patients are recruited into the study
Hepatitis C diagnosis	None	None	None	None	Baseline only	Only collected during the screening phase when patients are recruited into the study

NIDA = National Institute on Drug Abuse; BUP/NLX = Buprenorphine/naloxone; SMM = standard medical management; EMM = enhanced medical management; PWUD = people who use drugs; DSM-IV = Diagnostic and Statistical Manual of Mental Disorders, fourth edition; DSM-V = Diagnostic and Statistical Manual of Mental Disorders, fifth edition; OUD = opioid use disorder; HIV = human immunodeficiency virus; HRQoL = health-related quality of life

### Descriptive statistics

Table 3 provides descriptive statistics on the demographic, behavioral, and treatment characteristics of study participants in the data analysis sample. These results show that there were relatively few data points where participants were receiving treatment with medications for OUD. Participants from study NCT00316277 had, on average, a shorter history of extra-medical opioid use and a lower prevalence of injecting drugs when compared to those in the other studies. We provide additional descriptive statistics (Tables D1 to D3) and figures (Figures D1 to D6) in Appendix D showing the different patterns of self-reported opioid use and treatment with medications for OUD to inform the choice of interactions between the main fixed effects variables.

**Table 3:** Demographic, behavioral, and treatment characteristics of study participants in the data analysis

Clinical trial identifier	Baseline characteristics								All observations		
	Data analysis sample size	Mean age (SD)	Sex (= male), *n* (%)	Race and ethnicity				Mean number of years of extra-medical opioid use (SD)	Reporting daily (non-medical) opioid use, *n* (%)	Ongoing treatment with medications, *n* (%)	Reporting drug injecting, *n* (%)
				NH White, *n* (%)	NH Black, *n* (%)	Hispanic (any) , *n* (%)	NH Other, *n* (%)				
NCT00032955	107	36.7 (9.7)	65/107 (61%)	61/107 (57%)	21/107 (20%)	19/107 (18%)	6/107 (6%)	8.9 (8.6)	70/233 (30%)	1/233 (0.4%)	120/233 (52%)
NCT00032968	224	38.8 (10.2)	163/224 (73%)	89/224 (40%)	81/224 (36%)	49/224 (22%)	5/224 (2%)	9.7 (9.6)	277/520 (53%)	5/520 (1%)	220/520 (42%)
NCT00067158	137	43.6 (9.3)	74/137 (54%)	38/137 (28%)	38/137 (28%)	52/137 (38%)	9/137 (7%)	15.3 (10.1)	339/445 (76%)	6/445 (1%)	68/445 (2%)
NCT00316277	653	33.2 (10.2)	392/653 (60%)	583/653 (89%)	19/653 (3%)	30/653 (5%)	20/653 (3%)	5.2 (4.7)	466/1255 (37%)	3/1255 (0.2%)	9/1255 (1%)
NCT01612169	86	47.4 (8.5)	41/86 (48%)	14/86 (16%)	48/86 (56%)	15/86 (17%)	8/86 (9%)	19.6 (11.9)	45/214 (21%)	62/214 (29%)	68/214 (32%)
NCT02032433	570	33.9 (9.6)	401/570 (70%)	372/570 (65%)	54/570 (9%)	93/570 (16%)	45/570 (8%)	8.5 (7.2)	311/1523 (20%)	166/1523 (11%)	627/1523 (41%)

SD = standard deviation; NH White = non-Hispanic White; NH Black = non-Hispanic Black; NH Other = non-Hispanic Other

### Statistical analysis of health state utility data

The results from the main regression analysis showed that, on average, an additional day of heroin use in the past 30 days equates to a reduction in HSUVs of 0.002. This was smaller than the 0.003 reduction associated with an additional day of using other opiates, although people reporting heroin use were more likely to report injecting as their main route of administration compared to people reporting the use of other opiates (62% versus 18%), which is associated with an additional 0.043 reduction in health state utility. Table 4 shows contrasts from the main regression analysis and Table E1 in Appendix E shows results from the same analysis, with outputs on the beta-binomial scale. An HIV positive diagnosis was associated with a 0.074 reduction in HSUVs. The contrasts for the variables relating to heroin use and other opiate use were both found to be statistically significant. The interaction terms associated with these variables are retained in the model, despite them not being statistically significant, given that their exclusion on the grounds of statistical significance could potentially bias the cost-effectiveness results. The regression coefficients for the dummy variables indicating whether injecting was the main route of administration and identifying whether individuals had an HIV positive diagnosis were both statistically significant. All model coefficients related to the receipt of medications for OUD treatment were statistically non-significant, along with the corresponding HSUV contrast estimate.

**Table 4:** Estimated Health State Utilities’ (HSU) effects associated with contrasts for the predictor variables

Variable	Contrast	Mean difference in HSU (95% CI)
Days of heroin use	+ 1 day	-0.0019 (95% CI [-0.0033,-0.0001])
Days of other opiate use	+ 1 day	-0.0032 (95% CI [-0.0045,-0.0020])
Days of methadone use	+ 1 day	-0.0018 (95% CI [-0.0032,-0.0004])
Medications for opioid use disorder	Yes vs. no	-0.0096 (95% CI [-0.0673,0.0481])
Injecting as the main route of administration	Yes vs. no	-0.0425 (95% CI [-0.0792,-0.0057])
Human immunodeficiency virus (HIV)	Positive vs. no diagnosis	-0.0740 (95% CI [-0.1427,-0.0054])
Age	+ 1 year	-0.0014 (95% CI [-0.0025,-0.0002])

HSUV predictions from the main regression analysis show that uncertainty surrounding estimates is much higher for predictor combinations involving medications for OUD treatment compared to those without medications for OUD treatment. This uncertainty may be partly attributed to the small number of data points that were collected in people receiving medications for OUD treatment. Table 5 shows HSUV predictions for different combinations of predictor values considered to be relevant to OUD models. We specify multiple scenarios to reflect the possibility of extra-medical opioid use occurring, at a reduced frequency, alongside medications for OUD treatment, which are denoted by the health states numbered 3, 4, 9, and 10.

**Table 5:** Predicted Health State Utility (HSU) values for health states of interest in opioid use disorders (OUD) models

Health state number	Days of heroin use	Days of other opiate use	Injecting as the main route of administration	Receipt of MOUD treatment	HIV positive diagnosis	Mean HSU estimate (95% CI)
1	30	0	Yes	No	No	0.799 (95% CI [0.763,0.830])
2	0	30	No	No	No	0.817 (95% CI [0.786,0.845])
3	15	0	Yes	Yes	No	0.811 (95% CI [0.738,0.867])
4	0	15	No	Yes	No	0.843 (95% CI [0.746,0.907])
5	0	0	No	No	No	0.875 (95% CI [0.854,0.893])
6	0	0	No	Yes	No	0.881 (95% CI [0.809,0.928])
7	30	0	Yes	No	Yes	0.713 (95% CI [0.620,0.790])
8	0	30	No	No	Yes	0.737 (95% CI [0.644,0.812])
9	15	0	Yes	Yes	Yes	0.729 (95% CI [0.616,0.817])
10	0	15	No	Yes	Yes	0.771 (95% CI [0.636,0.865])
11	0	0	No	No	Yes	0.815 (95% CI [0.748,0.866])
12	0	0	No	Yes	Yes	0.824 (95% CI [0.726,0.891])

MOUD = medications for opioid use disorders; HIV = human immunodeficiency virus. Health state descriptions: (1) daily heroin use (injecting), no MOUD, and no HIV positive diagnosis; (2) daily opioid use (non-injecting and non-heroin), no MOUD, and no HIV positive diagnosis; (3) reduced/non-daily heroin use (injecting), MOUD, and no HIV positive diagnosis; (4) reduced/non-daily opioid use (non-injecting and non-heroin), MOUD, and no HIV positive diagnosis; (5) no extra-medical opioid use for 30 days, no MOUD, and no HIV positive diagnosis; (6) no extra-medical opioid use for 30 days, MOUD, and no HIV positive diagnosis; (7) daily heroin use (injecting), no MOUD, and a positive diagnosis for HIV; (8) daily opioid use (non-injecting and non-heroin), no MOUD, and a positive diagnosis for HIV; (9) reduced/non-daily heroin use (injecting), MOUD, and a positive diagnosis for HIV; (10) reduced/non-daily opioid use (non-injecting and non-heroin), MOUD, and a positive diagnosis for HIV; (11) no extra-medical opioid use for 30 days, no MOUD, and a positive diagnosis for HIV; (12) no extra-medical opioid use for 30 days, MOUD, and a positive diagnosis for HIV

**Fig. 2:**
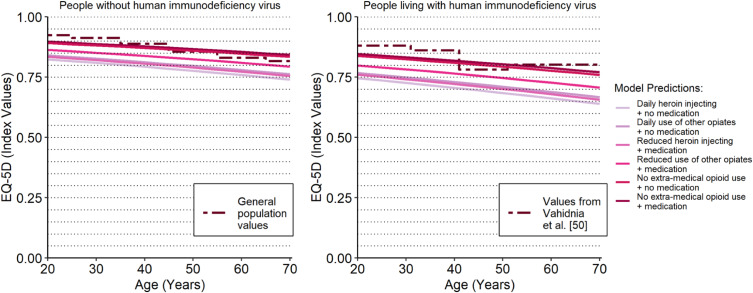
health state utility prediction from main analysis. The solid lines correspond to the various health states of interest in OUD cost-effectiveness models. The dotted lines reflect HSUV estimates from the published literature for the general population in the plot on the left and for populations living with HIV in the plot on the right

We found that age-adjusted HSUV predictions for the health states with no extra-medical opioid use were very similar to age-adjusted HSUV norms for the general population and for people living with HIV from the published literature [56, 57]. This finding is illustrated in Fig. 2, which shows the mean HSUV predictions for each of the health states specified in Table 5 with additional adjustments for age. The dashed lines represent the age-adjusted HSUV norms (i.e. mean values) for general and populations living with HIV from the published literature. As such, predictions under the dashed lines fall below the age-adjusted norms. The HSUV predictions for people not engaging in extra-medical opioid use increasingly exceed HSUV norms among the higher age groups. The generalizability of the predictions for people older than 60 years old is doubtful given that this age group constituted only 1% of the sample data.

In the sensitivity analysis to explore differences in outcomes across the studies, the model coefficients for study membership were statistically non-significant. Furthermore, the inclusion of study-specific dummy variables did not yield any changes in the mean predicted HSUVs larger than 0.031. Results from the sensitivity analysis can be found in Appendix F.

The results from the secondary analysis suggest that withdrawal symptoms are a fundamental driver of health state utilities in people with OUD. The coefficient for the SOWS measure was found to be statistically significant and a one-point change on this measure was associated with a 0.0049 reduction in HSUV (results in Table 6). The coefficients for the other predictors were all statistically non-significant (results shown in Table E2 in Appendix E). The HSUV predictions from the secondary analysis exhibit much more variability than those from the main analysis. Table 7 shows HSUV predictions from the secondary analysis with stratification of predictions according to different levels of heroin use and different categories of withdrawal symptoms (where the rows labelled A, B, and C refer to moderate, severe, and worst possible symptoms).

**Table 6:** Estimated Health State Utilities’ (HSU) effects associated with contrasts for the predictor variables in the secondary analysis

Variable	Contrast	Mean difference in HSU (95% CI)
Days of heroin use	+ 1 day	-0.0010 (95% CI [-0.0031, 0.0011])
Days of other opiate use	+ 1 day	-0.0024 (95% CI [-0.0054, 0.0006])
Days of methadone use	+ 1 day	-0.0018 (95% CI [-0.0178, 0.0137])
Medications for opioid use disorder	Yes vs. no	0.0131 (95% CI [-0.0479, 0.0769])
Injecting as the main route of administration	Yes vs. no	-0.0119 (95% CI [-0.0641, 0.0378])
Age	+ 1 year	-0.0004 (95% CI [-0.0026, 0.0016])
Subjective opioid withdrawal scale	+ 1 point on scale	-0.0049 (95% CI [-0.0070, -0.0039])

**Table 7:** Predicted Health State Utility (HSU) values for health states while controlling for subjective opiate withdrawal scale (SOWS) score

Health state number	Days of heroin use	Days of other opiate use	Injecting as the main route of administration	Receipt of MOUD treatment	SOWS score	Mean HSU estimate (95% CI)
1 – A	30	0	Yes	No	17	0.780 (95% CI [0.718, 0.830])
2 – A	0	30	No	No	17	0.763 (95% CI [0.628, 0.859])
3 – A	15	0	Yes	Yes	17	0.822 (95% CI [0.718, 0.893])
4 – A	0	15	No	Yes	17	0.804 (95% CI [0.644, 0.901])
5 – A	0	0	No	No	17	0.817 (95% CI [0.768, 0.858])
6 – A	0	0	No	Yes	17	0.877 (95% CI [0.715, 0.952])
1 – B	30	0	Yes	No	32	0.653 (95% CI [0.566, 0.729])
2 – B	0	30	No	No	32	0.631 (95% CI [0.460, 0.769])
3 – B	15	0	Yes	Yes	32	0.712 (95% CI [0.565, 0.823])
4 – B	0	15	No	Yes	32	0.686 (95% CI [0.473, 0.837])
5 – B	0	0	No	No	32	0.705 (95% CI [0.607, 0.786])
6 – B	0	0	No	Yes	32	0.793 (95% CI [0.559, 0.918])
1 – C	30	0	Yes	No	64	0.306 (95% CI [0.162, 0.473])
2 – C	0	30	No	No	64	0.283 (95% CI [0.104, 0.509])
3 – C	15	0	Yes	Yes	64	0.376 (95% CI [0.173, 0.600])
4 – C	0	15	No	Yes	64	0.344 (95% CI [0.110, 0.623])
5 – C	0	0	No	No	64	0.367 (95% CI [0.180, 0.575])
6 – C	0	0	No	Yes	64	0.493 (95% CI [0.178, 0.780])

MOUD = medications for opioid use disorders. Health state descriptions: (A-1) daily heroin use (injecting), no MOUD, no HIV positive diagnosis, and moderate withdrawal symptoms; (A-2) daily opioid use (non-injecting non-heroin), no MOUD, no HIV positive diagnosis, and moderate withdrawal symptoms; (A-3) reduced/non-daily heroin use (injecting), MOUD, no HIV positive diagnosis, and moderate withdrawal symptoms; (A-4) reduced/non-daily opioid use (non-injecting non-heroin), MOUD, no HIV positive diagnosis, and moderate withdrawal symptoms; (A-5) no extra-medical opioid use for 30 days, no MOUD, no HIV positive diagnosis, and moderate withdrawal symptoms; (A-6) no extra-medical opioid use for 30 days, MOUD, no HIV positive diagnosis, and moderate withdrawal symptoms; (B-1) daily heroin use (injecting), no MOUD, no HIV positive diagnosis, and severe withdrawal symptoms; (B-2) daily opioid use (non-injecting non-heroin), no MOUD, no HIV positive diagnosis, and severe withdrawal symptoms; (B-3) reduced/non-daily heroin use (injecting), MOUD, no HIV positive diagnosis, and severe withdrawal symptoms; (B-4) reduced/non-daily opioid use (non-injecting non-heroin), MOUD, no HIV positive diagnosis, and severe withdrawal symptoms; (B-5) no extra-medical opioid use for 30 days, no MOUD, no HIV positive diagnosis, and severe withdrawal symptoms; (B-6) no extra-medical opioid use for 30 days, MOUD, no HIV positive diagnosis, and severe withdrawal symptoms; (C-1) daily heroin use (injecting), no MOUD, no HIV positive diagnosis, and worst possible withdrawal symptoms; (C-2) daily opioid use (non-injecting non-heroin), no MOUD, no HIV positive diagnosis, and worst possible withdrawal symptoms; (C-3) reduced/non-daily heroin use (injecting), MOUD, no HIV positive diagnosis, and worst possible withdrawal symptoms; (C-4) reduced/non-daily opioid use (non-injecting non-heroin), MOUD, no HIV positive diagnosis, and worst possible withdrawal symptoms; (C-5) no extra-medical opioid use for 30 days, no MOUD, no HIV positive diagnosis, and worst possible withdrawal symptoms; (C-6) no extra-medical opioid use for 30 days, MOUD, no HIV positive diagnosis, and worst possible withdrawal symptoms

## Discussion

The results in this study show that HSUVs estimated among people with OUD are negatively associated with increased extra-medical opioid use, drug injecting, HIV positive diagnosis and increased age. The observed associations between HSUVs and treatment with medications for OUD are not statistically significant. However, HRQoL gains from medications for OUD treatment could still be achieved indirectly in a cost-effectiveness model through reductions in non-medicated opioid use or from reductions in the transmission of HIV. Regardless of these mechanisms for HRQoL gains, previous studies have shown that overdose mortality is the primary driver of QALY calculations in OUD rather than changes in HRQoL [18, 34].

This study expands on previous research by using responses from patients experiencing the key health states of interest rather than through vignette studies conducted in samples of the general population [6, 8]. The HSUVs estimated in this study are higher than those for equivalent health states derived in the vignette studies. For instance, HSUV predictions from the main analysis were 0.799 for people without HIV who inject heroin on a daily basis, compared to mean estimates of 0.588 and 0.574 in the studies by Connock et al. and Wittenberg et al., respectively [6, 8]. The lower estimates observed in the vignette studies may be attributable to the use of condition-specific labels in the valuation exercises, as opposed to the “generic” health dimensions of the EQ-5D-3 L.

### Strengths and limitations

One advantage of estimates in this study is that they reflect values from the US EQ-5D-3 L tariff and facilitate the comparable measurement of health benefits across studies using this instrument. This comparability helps to promote consistency in the use of cost-effectiveness evidence to inform policy decisions [58], which is especially important given the popularity of the EQ-5D [59]. Some further strengths of this study include its alignment with the needs of cost-effectiveness models in OUD, the systematic effort to make comprehensive use of relevant evidence in keeping with the principles of evidence-based medicine [60], and the use of a generic preference-based HSUV measure that has been validated in OUD [38].

One limitation of our study was that the data did not permit a delineation of the different phases of treatment with medications for OUD (i.e. induction/starts, stabilization, and maintenance), which could have important implications for HRQoL. Our results found that HSUV predictions involving treatment with medications for OUD varied depending on the level of extra-medical opioid use. Previous research has shown that the gains in HRQoL associated with medications for OUD treatment are modest over the short-term [61] and do not persist over the long-term [62]. Another study found a stronger negative association between increased opioid use (non-medicated) and social domains of HRQoL when participants were enrolled in treatment compared to those who had discontinued treatment [63].

The outputs from this work may also be inadequate for researchers requiring some specific alternative preference-based measure, such as the EQ-5D-5 L, and additional mappings would be required to produce estimates for such a measure. It is important to acknowledge the potential drawbacks of this study in using responses from patients experiencing the key health states of interest. With any health condition, there is potential for patients to adapt to their health state and, consequently, their responses to HRQoL questionnaires may change over time [64, 65]. Whether adaptation should be viewed as a concern is disputed as the argument has been used both for and against the use of experienced responses [66]. Another limitation is that the representation of drug types (i.e. heroin and ‘other opiates’) may be considered outdated given that the illegal supply of opioids is increasingly composed of synthetic variants, such as fentanyl. These new variants have been found to induce different forms of craving and withdrawal compared to heroin, as well as presenting new challenges in treatment with medications for OUD [67–69]. This shortcoming points to the need for data collection in patients using synthetic opioids to quantify their impacts on HRQoL. Our study also lacked the data needed to estimate statistical relationships between HSUVs and hepatitis C virus status, a highly prevalent infection among people who inject drugs. Similarly, our study lacked data related to incarceration status, pregnancies, birth outcomes, and hypoxia, which are potentially relevant states in cost-effectiveness models for OUD.

The findings are limited due to the reliance on trial data. Research has shown that participants from diverse or marginalized backgrounds are underrepresented in treatment trials [70]. The relevance of trial results can also be compromised by a ‘comorbidity gap’, when the incidence of comorbidities is underrepresented in trial participants when compared to the real-world population of interest [71]. This issue might explain why the age-specific HSUV predictions in Fig. 2 do not align with the HSUV norms for people aged fifty and older given that comorbidities disproportionately affect the recruitment of older participants into clinical trials [72]. The use of evidence from patient registries has received increased interest as researchers strive to incorporate data with more representative patient populations and routine care pathways [73, 74]. As such, this may represent a practical solution to ensure that future studies include a representative case mix of participants.

### Implications for future research

This study provides crucial evidence inputs for the estimation of QALYs in economic evaluations of interventions for OUD in a US setting. Ultimately, these estimates could give rise to different cost-effectiveness results and different policy decisions compared to the previous vignette studies because of the higher values for equivalent health states. Findings from the secondary analysis indicate that the explicit measurement of withdrawal symptoms in cost-effectiveness models might be an important line of pursuit for future research. The notion also relates to a broader research question to understand how measures of anhedonia, which is the inability to experience pleasure and one of the main characteristics of opioid withdrawal, might contribute to the development of evidence-based practices in the treatment in OUD [75]. The standard methodology in previous modeling studies has been to select intermediate endpoints – typically treatment retention and extra-medical drug use, both of which can be commonly found in clinical trials – to estimate the costs and QALYs associated with alternative policies [5, 16, 17]. This pragmatic approach to model development does not guarantee a conceptual model that accurately represents the experiences of people with lived experience of the condition under evaluation, which can lead to a misrepresentation of the key decision points [76]. This finding echoes calls for researchers to actively engage people with lived experience to ensure the models provide an appropriate representation of their “preferences, expectations and expanded definitions of what constitutes “successful” outcomes” [77, 78].

## Conclusion

This study presents an analysis of HSUV data from patients experiencing key OUD health states in six clinical trials for cost-effectiveness models of OUD. We observed that HSUVs were negatively associated with increased extra-medical opioid use, drug injecting, an HIV positive diagnosis and increased age. The results yielded HSUVs that were higher than those conducted in vignette studies, where samples of the general population were asked to value descriptions of defined OUD health states.

## Electronic supplementary material

Below is the link to the electronic supplementary material.

Supplementary Material 1

Supplementary Material 2
