# Supplementary material for: Analyzing quality of life among people with opioid use disorder from the National Institute on Drug Abuse Data Share initiative: implications for decision making
Source: Qual Life Res. Author manuscript; Available in PMC 2024 Oct 7. (PMC11452457; doi:10.1007/s11136-024-03729-6)
Supplement: Appendix [file NIHMS2025030-supplement-Appendix.docx]

**Appendices for study ‘Analyzing quality of life among people with opioid use disorder from the National Institute on Drug Abuse Data Share initiative: implications for decision making’**

**Contents**

[APPENDIX A: SUMMARY STATISTICS FOR TRIAL DATASETS 2](#_Toc150769632)

[**Table A1**: Selection of data points for the data analysis 4](#_Toc150769633)

[**Table A2:** Levels of missing data in the SF12 Version 1 measure across NIDA studies 5](#_Toc150769634)

[**Table A3:** Levels of missing data in the SF12 Version 2 measure across NIDA studies 5](#_Toc150769635)

[**Table A4:** Levels of missing data in the EQ-5D measure across NIDA studies 5](#_Toc150769636)

[APPENDIX B: OVERVIEW OF STUDY METHODOLOGY 6](#_Toc150769637)

[**Figure B1:** Flow diagram showing study steps 6](#_Toc150769638)

[APPENDIX B: MAPPING BETWEEN SF-12 VERSION 1 AND EQ-5D-3L 7](#_Toc150769639)

[**Table B1:** Missing data patterns in sample responding to both the EQ-5D-3L and the SF-12 Version 1 9](#_Toc150769640)

[**Table B2:** EQ-5D-3L responses from the 2001 Medical Expenditure Panel Survey by levels of missingness on the SF-12 Version 1 11](#_Toc150769641)

[**Table B3:** SF-12 Version 1 responses from the 2001 Medical Expenditure Panel Survey by levels of missingness on the EQ-5D-3L 12](#_Toc150769642)

[**Figure B1:** Graphical representation of the unidimensional model 15](#_Toc150769643)

[**Figure B2:** Graphical representation of the bifactor model 16](#_Toc150769644)

[**Table B4:** Sample Demographic Characteristics 18](#_Toc150769645)

[**Table B5:** Model Outputs for the Unidimensional and Bifactor Models 19](#_Toc150769646)

[**Table B6:** Model Fit Statistics for the Unidimensional and Bifactor Models 21](#_Toc150769647)

[**Figure B3:** Scatter Plot of Observed and Predicted EQ-5D-3L Values where the blue dots reflect predictions from the unidimensional LVM and the pink dots reflect predictions from the bifactor LVM. The diagonal line is included to show the point at which predicted and observed EQ-5D-3L would be equivalent 21](#_Toc150769648)

[**Table B7:** Mean absolute errors (95% confidence intervals) 22](#_Toc150769649)

[**Table B8:** Root mean squared errors (95% CI) 22](#_Toc150769650)

[**Figure B4:** Histogram of absolute prediction errors 23](#_Toc150769651)

[APPENDIX C: MAPPING BETWEEN SF-12 VERSION 2 AND EQ-5D-3L 28](#_Toc150769652)

[**Table C1:** Missing data patterns in sample responding to both the EQ-5D-3L and the SF-12 Version 2 30](#_Toc150769653)

[**Table C2:** EQ-5D-3L responses by levels of missingness on the SF-12 Version 2 31](#_Toc150769654)

[**Table C3:** SF-12 Version 2 responses by levels of missingness on the EQ-5D-3L 32](#_Toc150769655)

[**Table C4:** Sample Demographic Characteristics 35](#_Toc150769656)

[**Table C5:** Model Outputs for the Unidimensional and Bifactor Models 36](#_Toc150769657)

[**Table C5:** Sample Demographic Characteristics 38](#_Toc150769658)

[**Figure C1:** Scatter Plot of Observed and Predicted EQ-5D-3L Values where the blue dots reflect predictions from the unidimensional LVM and the pink dots reflect predictions from the bifactor LVM. The diagonal line is included to show the point at which predicted and observed EQ-5D-3L would be equivalent. 38](#_Toc150769659)

[**Table C6:** Mean absolute errors (95% confidence intervals) 39](#_Toc150769660)

[**Table C7:** Root mean squared errors (95% confidence intervals) 39](#_Toc150769661)

[**Figure C2:** Histogram of Absolute Prediction Errors 40](#_Toc150769662)

[APPENDIX D: ADDITIONAL SUMMARY STATISTICS FOR TRIAL DATASETS 45](#_Toc150769663)

[**Figure D1:** Histograms displaying responses to the self-reported number of days of heroin use conditional on the self-reported number of days using other opiates 46](#_Toc150769664)

[**Figure D2:** Histograms displaying responses to the self-reported number of days of heroin use conditional on the self-reported number of days using methadone 47](#_Toc150769665)

[**Figure D3:** Histograms displaying responses to the self-reported number of days of other opiate use conditional on the self-reported number of days using methadone 48](#_Toc150769666)

[**Table D1:** Frequency distribution displaying responses to the self-reported number of days of heroin use and treatment status with medications for opioid use disorder (MOUD) 49](#_Toc150769667)

[**Figure D4:** Histogram displaying responses to the self-reported number of days of heroin use conditional on treatment status with medications for opioid use disorder (MOUD) 49](#_Toc150769668)

[**Table D2:** Frequency distribution displaying responses to the self-reported number of days of other opiate use and treatment status with medications for opioid use disorder (MOUD) 50](#_Toc150769669)

[**Figure D5:** Histogram displaying responses to the self-reported number of days of other opiate use conditional on treatment status with medications for opioid use disorder (MOUD) 50](#_Toc150769670)

[**Table D3:** Frequency distribution displaying responses to the self-reported number of days of methadone use and treatment status with medications for opioid use disorder (MOUD) 51](#_Toc150769671)

[**Figure D6:** Histogram displaying responses to the self-reported number of days of methadone use conditional on treatment status with medications for opioid use disorder (MOUD) 51](#_Toc150769672)

[APPENDIX E: RESULTS FROM REGRESSION ANALYSES 52](#_Toc150769673)

[**Table E1:** Results from the main analysis on the beta-binomial scale 52](#_Toc150769674)

[**Table E2:** Results from the secondary analyses on the beta-binomial scale 52](#_Toc150769675)

[APPENDIX F: RESULTS FROM SENSITIVITY ANALYSIS 53](#_Toc150769676)

[**Table F1:** Results from the regression sensitivity analysis on the beta-binomial scale 54](#_Toc150769677)

[**Table F2:** Estimated Health State Utilities’ (HSU) effects associated with contrasts for the predictor variables from the regression sensitivity analysis 55](#_Toc150769678)

[**Table F3:** Predicted Health State Utility (HSU) values for health states of interest in opioid use disorders (OUD) models 56](#_Toc150769679)

[References 57](#_Toc150769680)

# APPENDIX A: SUMMARY STATISTICS FOR TRIAL DATASETS

Table A1 shows the number of participants enrolled and randomized to treatments (i.e. meeting inclusion criteria) in the trials selected for analysis. Distinctions are made between the total number of participants included within each study and the number meeting the diagnostic criteria for either opioid dependence or opioid use disorder (depending on which version of the Diagnostic and Statistical Manual of Mental Disorders was used). These data are based on information documented previously [1–5]. Table A1 also provides a breakdown of the number of observations that needed to be dropped for one of the following reasons: (i) a lack of alignment between variables in the timing of the collection of the data; (ii) urine drug screen results that contradicted individuals’ responses to the self-reported drug use questions; or (iii) data were missing and there was no accompanying data that would facilitate imputation. Tables A2 to A4 show the levels of missing health-related quality of life (HRQoL) data across the various National Institute on Drug Abuse (NIDA) studies included in this analysis, having already removed observations with missing data across all items. The observations with partially missing data are included in the analysis sample because the latent variable model (LVM) approach to mapping can estimate the latent factor score for these observations based on the items where data are available.

## **Table A1**: Selection of data points for the data analysis

| **Clinical trial identifier** | **Sample size** | | | **Total observations^1^** | **Observations dropped** | | | | **Number of observations in analysis** |
| --- | --- | --- | --- | --- | --- | --- | --- | --- | --- |
|  | **Randomized** | **Meeting OUD inclusion criteria** | **Analysis** |  | **No response to HRQoL measure** | **Urine Drug Screen contradicts self-reported opioid use** | **Misalignment in the timing of data collection across variables** | **Missing self-report opioid responses** |  |
| NCT00032955 | 113 | 113 | 107 | 250 | 1 | 2 | 13 | 1 | 233 |
| NCT00032968 | 230 | 230 | 224 | 559 | 7 | 10 | 19 | 3 | 520 |
| NCT00067158 | 415 | 139 | 137 | 474 | 2 | 5 | 17 | 5 | 445 |
| NCT00316277 | 653 | 653 | 653 | 1,387 | 73 | 4 | 21 | 34 | 1,255 |
| NCT01612169 | 801 | 89 | 86 | 240 | 0 | 4 | 22 | 2 | 214 |
| NCT02032433 | 570 | 570 | 570 | 1,547 | 5 | 9 | 3 | 7 | 1,523 |

^1^ These numbers reflect the data at time points where both self-reported HRQoL and drug use questionnaires were administered.

OUD = Opioid use disorder; HRQoL = health-related quality of life.

## **Table A2:** Levels of missing data in the SF12 Version 1 measure across NIDA studies

|  |  | **NCT00032955** | **NCT00032968** | **NCT00067158** |
| --- | --- | --- | --- | --- |
| **Number of items with missing data** | **0** | 218 | 488 | 441 |
|  | **1** | 12 | 29 | 5 |
|  | **2** | 3 | 3 | 1 |
|  | **4** | 0 | 2 | 0 |
|  | **6** | 0 | 0 | 1 |
| **Column totals** | | 233 | 522 | 448 |

NIDA = National Institute on Drug Abuse

## **Table A3:** Levels of missing data in the SF12 Version 2 measure across NIDA studies

|  |  | **NCT00316277** | **NCT01612169** |
| --- | --- | --- | --- |
| **Number of items with missing data** | **0** | 1,255 | 212 |
|  | **1** | 0 | 1 |
|  | **5** | 0 | 1 |
| **Column totals** | | 1,255 | 214 |

NIDA = National Institute on Drug Abuse

## **Table A4:** Levels of missing data in the EQ-5D measure across NIDA studies

|  |  | **NCT02032433** |
| --- | --- | --- |
| **Number of items with missing data** | **0** | 1,522 |
|  | **1** | 1 |
| **Column totals** | | 1,523 |

NIDA = National Institute on Drug Abuse

# APPENDIX B: MAPPING BETWEEN SF-12 VERSION 1 AND EQ-5D-3L

**Title**

Mapping responses to the SF-12 Version 1 onto responses to the EQ-5D-3L using survey data collected in a sample of the United States (U.S.) population

**Study Rationale**

The rationale for developing mapping algorithms in this paper is to support the comprehensive use of health-related quality of life (HRQoL) evidence from the National Institute on Drug Abuse (NIDA) Data Share initiative to represent the health burden associated with health states typically found in opioid use disorder (OUD) models in the published literature. This approach avoids disregarding potentially relevant evidence, simply because it has not been produced using a specific HRQoL measure.

**Study Objective**

The purpose of this analysis is to develop a mapping algorithm linking the SF-12 Version 1 (source measure) and the EQ-5D-3L (target measure) using data from the 2001 Medical Expenditure Panel Survey (MEPS), which is a nationally representative survey of the non-institutionalized U.S. population [6]. The development of this algorithm is intended to facilitate the prediction of EQ-5D-3L responses using SF-12 Version 1 data collected in trials identified through the NIDA Data Share initiative and the estimation of health index values reflecting U.S. population-based preference weights [7].

**Estimation Sample**

For SF-12 Version 1 and EQ-5D-3L, data were obtained from the Household Component of the 2001 MEPS. This data has been used in previous mapping studies linking these measures [8, 9]. The 2001 MEPS data provides a nationally representative indication of health care use, expenditures, sources of payment, and health insurance coverage for the U.S. civilian non-institutionalized population. In 2001, the Household Component of MEPS also provided estimates of respondents' demographic and socio-economic characteristics, as well as their self-reported health status measured through the SF-12 Version 1 and the EQ-5D-3L [10]. The sampling frame for the MEPS HC was drawn from respondents to National Health Interview Survey, which was based on a stratified multistage sample design [11], and included responses from individuals located throughout the United States. The rationale for its use as the estimation sample was twofold: (i) the data are publicly available online (from <https://www.meps.ahrq.gov/>), and (ii) it provides a nationally representative survey of the non-institutionalized U.S. population.

**External Validation Sample**

To test the external validity of the mapping algorithm developed, data were obtained from the Household Component of the 2000 MEPS. The 2000 MEPS study design was similar to that in the 2001 MEPS study, and it also collected both SF-12 Version 1 and EQ-5D-3L measures [10]. There is partial overlap between MEPS samples across successive years and, as such, the data from 2000 and 2001 were not combined for the estimation of the mapping algorithm to avoid bias caused by unobservable correlations between sample observations. However, this meant that the 2000 MEPS data could be used to validate the predictive performance of the mapping algorithm developed using the 2001 MEPS data.

**Source and Target Measures**

The EQ-5D-3L was designated as the target measure for valuing HRQoL in this study following previous research indicating it to be the preferable generic, indirect utility measure [12]. The EQ-5D-3L has two components [13]: (i) a descriptive system comprised of five dimensions (mobility, self-care, usual activities, pain/discomfort, and anxiety/depression), each of which has 3 levels (no problems, some problems, and extreme problems), and (ii) a value set reflecting preferences for the different health states within the descriptive system among a sample of the jurisdiction of interest. The source measure of HRQoL in the mapping exercises was the SF-12 Version 1, which is a self-reported outcome measure assessing the impact of health on an individual’s everyday life [14]. The SF-12 Version 1 is composed of twelve questions relating to eight health domains (limitations in physical activities because of health problems, limitations in social activities because of physical or emotional problems, limitations in usual role activities because of physical health problems, bodily pain, general mental health, limitations in usual role activities because of emotional problems, vitality, and general health perceptions).

**Conceptual Overlap Between Measures**

There were no qualitative studies (e.g. item content review) identified in the published literature assessing the conceptual overlap between the EQ-5D-3L and SF-12 (Version 1). However, previous studies have provided empirical evidence to show the overlap in the constructs of the two measures. Two studies have demonstrated the convergent and divergent validity of the EQ-5D-3L based on the observed relationship between the responses to EQ-5D-3L dimensions and the SF-12 (Version 1) component scores [15, 16]. It should be noted, however, that both studies also identified an important ceiling effect of the EQ-5D-3L when comparing the two measures. One possible explanation for the latter finding may be that, unlike the SF-12, the EQ-5D-3L lacks any dimension corresponding to energy or vitality.

**Exploratory Data Analysis**

Of the 33,556 individuals in the Household Component of the 2001 MEPS survey, 19,358 individuals provided complete responses to all items of the SF-12 Version 1 and the EQ-5D. Table B1 provides a breakdown of the level of missing data for the two instruments. Table B2 shows EQ-5D responses across differing levels of missingness on the SF-12. The data show that as the number of missing items on the SF-12 increase, there are fewer people reporting no health problems on the EQ-5D dimensions. This trend cannot be seen among patients with more than 6 items missing on the SF-12 but this is likely to be attributable to the smaller sample sizes in these categories. Table B3 shows SF-12 responses across differing levels of missingness on the EQ-5D which shows a similar trend to Table B2, i.e. larger health burden in one measure associated with greater levels of missing data in the other measure.

## **Table B1:** Missing data patterns in sample responding to both the EQ-5D-3L and the SF-12 Version 1

|  | | **Number of EQ-5D items with missing data** | | | | | | **Row totals** |
| --- | --- | --- | --- | --- | --- | --- | --- | --- |
|  |  | **0** | **1** | **2** | **3** | **4** | **5** |  |
| **Number of SF-12 items with missing data** | **0** | 19,358 | 269 | 39 | 10 | 21 | 11 | 19,708 |
|  | **1** | 732 | 61 | 10 | 2 | 1 | 16 | 822 |
|  | **2** | 114 | 18 | 8 | 2 | 0 | 3 | 145 |
|  | **3** | 36 | 8 | 1 | 3 | 1 | 1 | 50 |
|  | **4** | 31 | 3 | 1 | 3 | 1 | 2 | 41 |
|  | **5** | 15 | 3 | 2 | 0 | 0 | 1 | 21 |
|  | **6** | 6 | 4 | 0 | 0 | 0 | 1 | 11 |
|  | **7** | 9 | 1 | 2 | 1 | 1 | 1 | 15 |
|  | **8** | 2 | 1 | 1 | 0 | 1 | 2 | 7 |
|  | **9** | 7 | 0 | 4 | 1 | 0 | 1 | 13 |
|  | **10** | 7 | 1 | 2 | 0 | 0 | 2 | 12 |
|  | **11** | 94 | 2 | 0 | 1 | 1 | 4 | 102 |
|  | **12** | 7 | 0 | 0 | 0 | 0 | 12,602 | 12,609 |
| **Column totals** | | 20,418 | 371 | 70 | 23 | 27 | 12,647 | 33,556 |

Observations were dropped if they were missing any more than 50% of items for either of the measurements (N=12,837). A hypothesis test [17] was performed on the remaining data, which showed that the data are not missing completely at random, i.e. p-value for the hypothesis that the data are Missing Completely At Random (MCAR) was 0. As such, the data were assumed to be Missing at Random (MAR) and multiple imputation were used to deal with the missing data [18]. This approach predicts a response for each item on the SF-12 Version 1 and EQ-5D-3L with missing variables conditional on the response to all other items available. No auxiliary variables were included. Five sets of imputed data were produced via multivariate imputation with chained equations. The mapping models (described in the next section) were then fitted to each of the imputed datasets and the outputs were then combined via pooling methods.

## **Table B2:** EQ-5D-3L responses from the 2001 Medical Expenditure Panel Survey by levels of missingness on the SF-12 Version 1

|  | Number of SF-12 items with missing data | | | | | | | | | | | | |
| --- | --- | --- | --- | --- | --- | --- | --- | --- | --- | --- | --- | --- | --- |
|  | 0 | 1 | 2 | 3 | 4 | 5 | 6 | 7 | 8 | 9 | 10 | 11 | 12 |
| Mobility = No problems | 81.6% | 71.7% | 66.7% | 58.3% | 51.6% | 46.7% | 33.3% | 77.8% | 100.0% | 57.1% | 42.9% | 70.2% | 71.4% |
| Mobility = Some problems | 18.0% | 27.5% | 28.9% | 38.9% | 48.4% | 46.7% | 66.7% | 22.2% | 0.0% | 42.9% | 57.1% | 28.7% | 14.3% |
| Mobility = Confined to bed | 0.4% | 0.8% | 4.4% | 2.8% | 0.0% | 6.7% | 0.0% | 0.0% | 0.0% | 0.0% | 0.0% | 1.1% | 14.3% |
| Self care = No problems | 95.5% | 91.8% | 86.0% | 86.1% | 83.9% | 80.0% | 66.7% | 100.0% | 50.0% | 71.4% | 57.1% | 91.5% | 85.7% |
| Self care = Some problems | 3.9% | 6.7% | 9.6% | 13.9% | 16.1% | 6.7% | 33.3% | 0.0% | 50.0% | 28.6% | 28.6% | 8.5% | 0.0% |
| Self care = Unable to wash/dress | 0.6% | 1.5% | 4.4% | 0.0% | 0.0% | 13.3% | 0.0% | 0.0% | 0.0% | 0.0% | 14.3% | 0.0% | 14.3% |
| Usual activities = No problems | 80.7% | 73.5% | 63.2% | 55.6% | 54.8% | 53.3% | 50.0% | 77.8% | 50.0% | 57.1% | 71.4% | 66.0% | 85.7% |
| Usual activities = Some problems | 17.3% | 22.0% | 28.1% | 30.6% | 35.5% | 33.3% | 33.3% | 22.2% | 50.0% | 42.9% | 14.3% | 29.8% | 0.0% |
| Usual activities = Unable to perform | 2.0% | 4.5% | 8.8% | 13.9% | 9.7% | 13.3% | 16.7% | 0.0% | 0.0% | 0.0% | 14.3% | 4.3% | 14.3% |
| Pain = None | 56.5% | 47.5% | 42.1% | 52.8% | 32.3% | 33.3% | 16.7% | 44.4% | 100.0% | 14.3% | 42.9% | 45.7% | 57.1% |
| Pain = Moderate | 39.8% | 44.9% | 50.0% | 33.3% | 61.3% | 46.7% | 66.7% | 55.6% | 0.0% | 71.4% | 42.9% | 44.7% | 28.6% |
| Pain = Extreme | 3.8% | 7.5% | 7.9% | 13.9% | 6.5% | 20.0% | 16.7% | 0.0% | 0.0% | 14.3% | 14.3% | 9.6% | 14.3% |
| Anxiety = None | 72.8% | 69.5% | 63.2% | 63.9% | 64.5% | 73.3% | 66.7% | 66.7% | 100.0% | 57.1% | 71.4% | 56.4% | 57.1% |
| Anxiety = Moderate | 24.7% | 27.6% | 31.6% | 30.6% | 25.8% | 20.0% | 33.3% | 33.3% | 0.0% | 42.9% | 28.6% | 40.4% | 28.6% |
| Anxiety = Extreme | 2.5% | 2.9% | 5.3% | 5.6% | 9.7% | 6.7% | 0.0% | 0.0% | 0.0% | 0.0% | 0.0% | 3.2% | 14.3% |

## **Table B3:** SF-12 Version 1 responses from the 2001 Medical Expenditure Panel Survey by levels of missingness on the EQ-5D-3L

|  | Number of EQ-5D items with missing data | | | | | |
| --- | --- | --- | --- | --- | --- | --- |
|  | 0 | 1 | 2 | 3 | 4 | 5 |
| General Health = Excellent | 16.3% | 12.3% | 2.6% | 0.0% | 14.3% | 27.3% |
| General Health = Very good | 36.5% | 23.8% | 23.1% | 30.0% | 33.3% | 27.3% |
| General Health = Good | 31.5% | 36.1% | 35.9% | 10.0% | 38.1% | 36.4% |
| General Health = Fair | 12.7% | 20.8% | 30.8% | 50.0% | 14.3% | 9.1% |
| General Health = Poor | 3.0% | 7.1% | 7.7% | 0.0% | 0.0% | 0.0% |
| Accomplish less (mental) = Yes | 18.6% | 27.5% | 33.3% | 50.0% | 4.8% | 9.1% |
| Accomplish less (mental) = No | 81.4% | 72.5% | 66.7% | 50.0% | 95.2% | 90.9% |
| Accomplish less (physical) = Yes | 22.7% | 37.2% | 51.3% | 50.0% | 23.8% | 0.0% |
| Accomplish less (physical) = No | 77.3% | 62.8% | 48.7% | 50.0% | 76.2% | 100.0% |
| Felt calm/peaceful = All the time | 12.1% | 13.8% | 7.7% | 0.0% | 14.3% | 27.6% |
| Felt calm/peaceful = Most of the time | 41.2% | 32.0% | 17.9% | 40.0% | 38.1% | 63.6% |
| Felt calm/peaceful = A good bit of the time | 19.0% | 17.1% | 20.5% | 10.0% | 23.8% | 0.0% |
| Felt calm/peaceful = Some of the time | 17.8% | 22.7% | 28.2% | 20.0% | 14.3% | 0.0% |
| Felt calm/peaceful = A little of the time | 7.5% | 10.0% | 17.9% | 20.0% | 4.8% | 0.0% |
| Felt calm/peaceful = None of the time | 2.4% | 4.5% | 7.7% | 10.0% | 4.8% | 0.0% |
| Limited moderate activities = Yes, limited a lot | 8.4% | 17.1% | 28.2% | 20.0% | 14.3% | 0.0% |
| Limited moderate activities = Yes, limited a little | 17.2% | 24.9% | 25.6% | 10.0% | 9.5% | 18.2% |
| Limited moderate activities = No, not limited at all | 74.5% | 58.0% | 46.2% | 70.0% | 76.2% | 81.8% |
| Felt downhearted/blue = All the time | 1.4% | 4.1% | 5.1% | 0.0% | 4.8% | 0.0% |
| Felt downhearted/blue = Most of the time | 3.6% | 4.5% | 0.0% | 30.0% | 0.0% | 0.0% |
| Felt downhearted/blue = A good bit | 5.6% | 7.8% | 0.0% | 20.0% | 4.8% | 0.0% |
| Felt downhearted/blue = Some of the time | 19.0% | 26.0% | 33.3% | 10.0% | 14.3% | 0.0% |
| Felt downhearted/blue = A little of the time | 36.9% | 31.6% | 35.9% | 20.0% | 23.8% | 72.7% |
| Felt downhearted/blue = None of the time | 33.5% | 26.0% | 25.6% | 20.0% | 52.4% | 27.3% |
| Limited work (mental) = Yes | 18.4% | 28.3% | 35.9% | 80.0% | 28.6% | 36.4% |
| Limited work (mental) = No | 81.6% | 71.7% | 64.1% | 20.0% | 71.4% | 63.6% |
| Limited work (physical) = Yes | 22.0% | 34.2% | 51.3% | 30.0% | 28.6% | 0.0% |
| Limited work (physical) = No | 78.0% | 65.8% | 48.7% | 70.0% | 71.4% | 100.0% |
| Lots of energy = All the time | 10.0% | 11.2% | 10.3% | 10.0% | 14.3% | 36.4% |
| Lots of energy = Most of the time | 39.2% | 30.5% | 15.4% | 20.0% | 28.6% | 36.4% |
| Lots of energy = A good bit of the time | 20.0% | 17.8% | 17.9% | 20.0% | 33.3% | 0.0% |
| Lots of energy = Some of the time | 18.9% | 24.2% | 33.3% | 20.0% | 14.3% | 9.1% |
| Lots of energy = A little of the time | 8.2% | 8.6% | 10.3% | 20.0% | 9.5% | 18.2% |
| Lots of energy = None of the time | 3.7% | 7.8% | 12.8% | 10.0% | 0.0% | 0.0% |
| Health stopped social activities = All of the time | 2.3% | 8.6% | 2.6% | 0.0% | 0.0% | 0.0% |
| Health stopped social activities = Most of the time | 4.2% | 6.7% | 5.1% | 40.0% | 0.0% | 18.2% |

**Table B3**: SF-12 Version 1 responses from the 2001 Medical Expenditure Panel S survey by levels of missingness on the EQ-5D-3L (continued)

|  | Number of EQ-5D items with missing data | | | | | |
| --- | --- | --- | --- | --- | --- | --- |
|  | 0 | 1 | 2 | 3 | 4 | 5 |
| Health stopped social activities = Some of the time | 12.4% | 16.7% | 28.2% | 20.0% | 9.5% | 0.0% |
| Health stopped social activities = A little of the time | 16.1% | 16.7% | 15.4% | 20.0% | 9.5% | 18.2% |
| Health stopped social activities = None of the time | 65.0% | 51.3% | 48.7% | 20.0% | 81.0% | 63.6% |
| Pain limits work = Not at all | 54.4% | 41.3% | 30.8% | 10.0% | 52.4% | 63.6% |
| Pain limits work = A little bit | 24.6% | 24.2% | 25.6% | 50.0% | 19.0% | 36.4% |
| Pain limits work = Moderately | 10.9% | 17.1% | 17.9% | 0.0% | 14.3% | 0.0% |
| Pain limits work = Quite a bit | 7.6% | 10.0% | 23.1% | 40.0% | 14.3% | 0.0% |
| Pain limits work = Extremely | 2.6% | 7.4% | 2.6% | 0.0% | 0.0% | 0.0% |
| Health limits climbing stairs = Yes, limited a lot | 10.0% | 16.4% | 28.2% | 0.0% | 9.5% | 9.1% |
| Health limits climbing stairs = Yes, limited a little | 20.9% | 29.4% | 35.9% | 40.0% | 23.8% | 18.2% |
| Health limits climbing stairs = No, not limited at all | 69.1% | 54.3% | 35.9% | 60.0% | 66.7% | 72.7% |

**Model specification for mapping algorithms**

All mapping algorithms were implemented within a latent variable modeling (LVM) framework by assuming that the Likert-type responses to HRQoL questions are manifestations of an underlying continuous scale (referred to here as the latent response scale). The theoretical basis for this approach is based on methodological insights from the field of psychometrics research and the assumption that the different patient reported outcome measures represent alternative realizations of the same underlying construct [19]. For a given item, each individual is assumed to lie somewhere on the latent response scale and it is the location on this scale that determines the observed category selected. Each of the latent response scales are assumed to have an associated distribution and the aim of the overall model is to investigate the extent to which a hypothesized latent factor accounts for correlations between these distributions using polychoric correlations [20]. The latent response scales are assumed to follow a normal distribution, where the area under the curve represents the proportion of patients realizing a given response level. Distributions are constructed for each of these scales by assigning threshold values defining the point at which people change their selected response category. These values typically represent points on the standard normal distribution and can be used to approximate the proportion of patients in each category using the cumulative distribution function.

To start with, we consider a model specification with a single latent factor defined by all the indicators across the two instruments as shown in Figure B1. The variability of each indicator is separated into two components: (1) the variation attributable to the latent factor, and (2) unexplained error variance, represented by the $\varepsilon_{i}$ terms. This model was fitted to the data using the lavaan package in R [21]. The model in Figure B1 assumes that the error terms are independent of one another. One might question the validity this assumption if there are concerns about multidimensionality especially in the presence of well-defined clusters of items. An alternative model specification that researchers might consider for scenarios involving multidimensionality in addition to a common trait is the bi-factor model [22]. This approach was employed in a previous study linking the SF-12 Version 1 and the EQ-5D-3L using the 2000 MEPS data [23]. The bifactor model posits that there are three sources of variability underlying the item responses attributable to: (i) a latent factor that explains variation in all items across the two measurements (‘Common Latent Factor’); (ii) measurement-specific latent factors (‘EQ-5D Latent Factor’ and ‘SF-12 Latent Factor’); (iii) unexplained error variance (terms $\varepsilon_{1}$ – $\varepsilon_{17}$). Figure B2 provides a graphical representation of the bifactor model linking SF-12 Version 1 and the EQ-5D-3L.

## **Figure B1:** Graphical representation of the unidimensional model


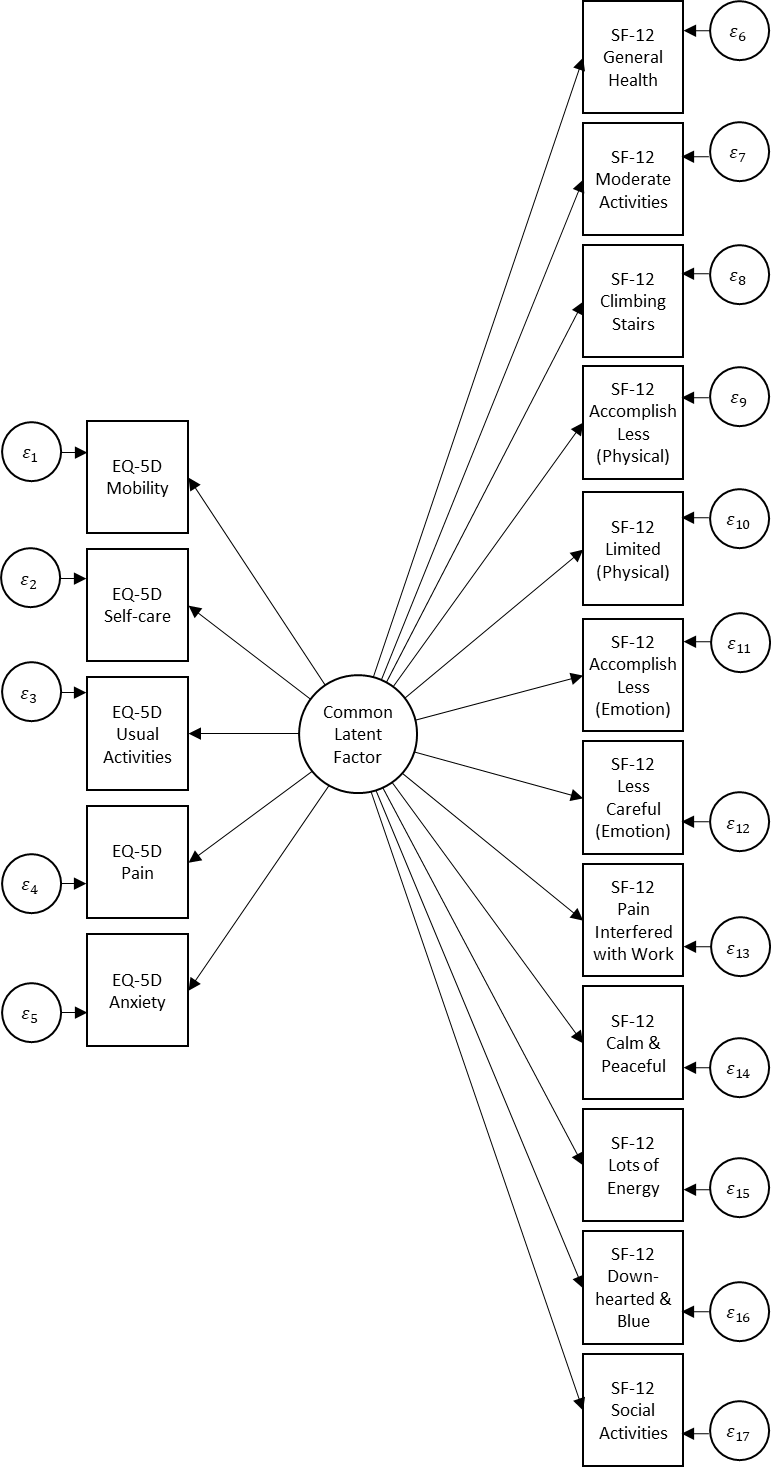


## **Figure B2:** Graphical representation of the bifactor model


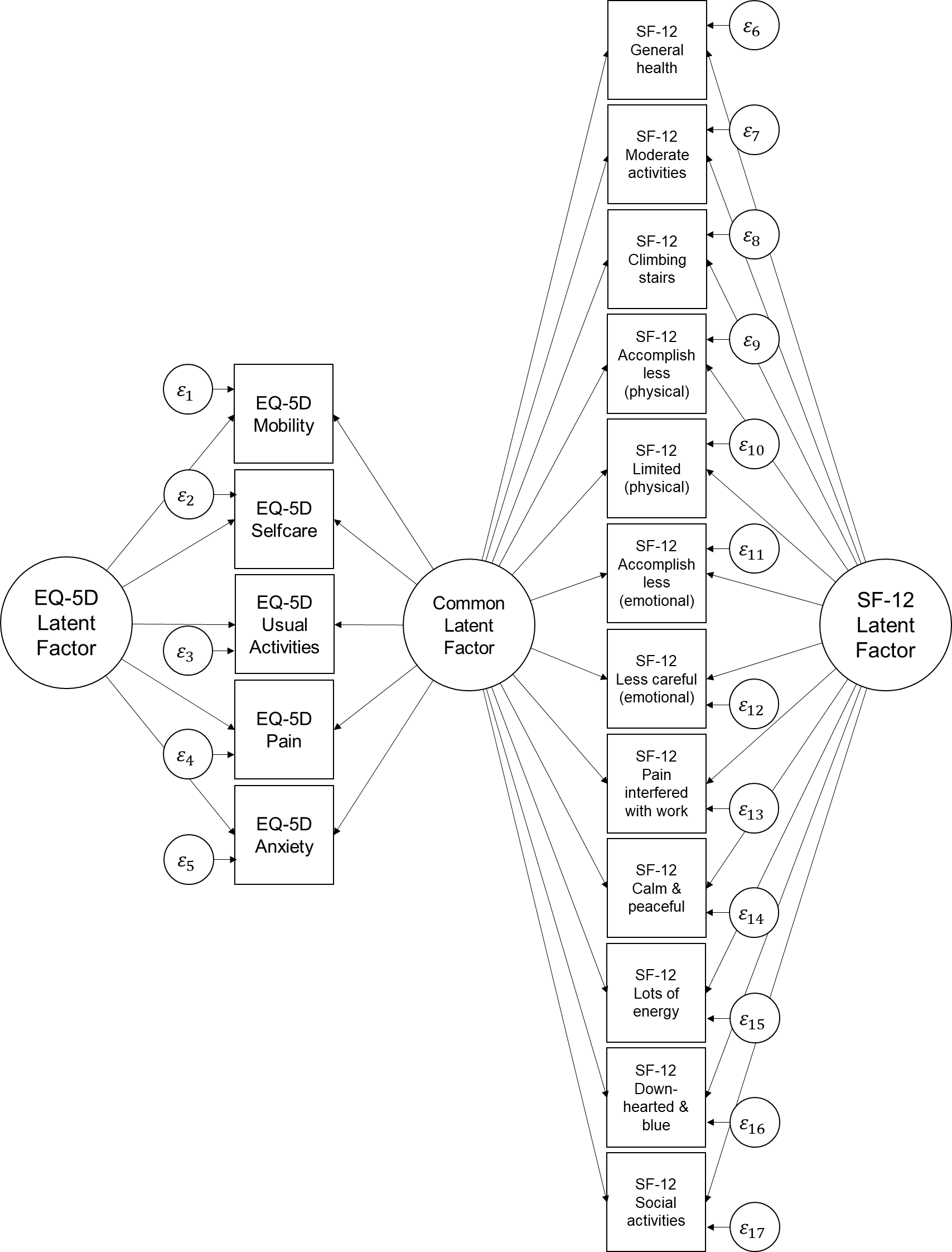


**Prediction of EQ-5D-3L Responses**

The process of predicting EQ-5D-3L responses using the mapping algorithms in this study was composed of several steps. The outputs from the LVM model were fitted to new data only containing the SF-12 Version 1. The LVM models had their parameter estimates fixed to those derived from the original mapping analysis and were also specified to have free parameters associated with the latent variable (mean and variance) in order to permit the estimation of factor scores. Once factor scores were calculated for the new data, they were used to predict the expected responses to the EQ-5D-3L items using the relevant factor loadings and threshold values from the original mapping analysis. Predictions were made using the expected utility method described in previous research [24].

**Validation of EQ-5D-3L Predictions and Measures of Model Performance**

Following recommended practice, the mapping algorithms were validated in a dataset, the 2000 MEPS data, different from the one used to generate it [25]. While there is partial overlap between this data and the 2001 MEPS data in terms of the sample participants, the responses were provided a year apart and thus still represent data that was external to that used to derive the mapping algorithms. Model performance was also assessed in terms of the differences between observed and predicted health index values by calculating the mean absolute error and the mean squared error terms. Probabilistic predictions were produced to account for the sample uncertainty surrounding the parameters in each of the mapping models using Monte Carlo sampling methods (50 simulations). The sampling process was based on 50 simulations, which was considered optimal when weighing up the precision of the uncertainty estimates against the amount of time it took to run these analyses (For reference, the uncertainty estimates generated with this many samples only varied at the fifth decimal point). The preferred mapping algorithm was selected on the basis of model fit and predictive performance [26].

**Results**

**Sample Description**

Twenty thousand seven hundred and nineteen individuals from the 2001 MEPS study provided the minimum number of items required for inclusion in the sample for analysis (i.e. 50% or more of the items per instrument). Out of this sample, 19,358 provided responses for all the EQ-5D-3L and SF-12 Version 1 items. Missing data were imputed using multivariate imputation by chained equations techniques [18]. The validation sample (2000 MEPS study) had twelve thousand nine hundred and ninety eight individuals providing complete responses for all the EQ-5D-3L and SF-12 Version 1 items. Table B4 provides descriptive statistics for the final data used in the mapping analysis and for the data used for the validation of the mapping algorithms.

## **Table B4:** Sample Demographic Characteristics

|  | 2001 MEPS | 2000 MEPS |
| --- | --- | --- |
| Age (years) |  |  |
| -Mean | 46 | 45 |
| -Lower quartile | 32 | 31 |
| -Median | 44 | 43 |
| -Upper quartile | 58 | 56 |
| -Minimum | 18 | 18 |
| -Maximum | 85 | 90 |
| Sex |  |  |
| -Female (%) | 54% | 53% |
| US Census Region |  |  |
| -Northeast (%) | 16% | 16% |
| -Midwest (%) | 22% | 22% |
| -South (%) | 38% | 38% |
| -West (%) | 25% | 24% |
| EQ-5D index values |  |  |
| -Mean | 0.862 | 0.865 |
| -Lower quartile | 0.800 | 0.800 |
| -Median | 0.844 | 0.844 |
| -Upper quartile | 1.000 | 1.000 |
| -Minimum | -0.109 | -0.109 |
| -Maximum | 1.000 | 1.000 |

MEPS = Medical Expenditure Panel Survey

**Model Outputs**

Table B5 shows the outputs from the unidimensional and bifactor models. A pooled variance covariance matrix was produced to capture the correlations between each of the model parameters when characterizing the sample uncertainty associated with the parameter estimates in the mapping algorithm. Following recommendations from Kline (2015), the following tests of model fit were performed: chi-squared test, the root mean square error of approximation (RMSEA), the comparative fit index (CFI), and the standardized root mean square residual (SRMR). The chi-squared test indicated a good model fit for both the unidimensional and bifactor models (p<0.05). The remaining test results are shown in Table B6. The RMSEA is a parsimony-adjusted index, where values >0.10 are regarded as being indicative of poor fit between the hypothesized model and the observed variance-covariance matrix. The CFI is a relative measure that compares the fit of a target model to the fit of an independent (or null) model, where values ≥ 0.95 have been proposed as representing an accepTable Eit [28]. The Tucker-Lewis index (TLI) compares the proposed factor model to a model in which no interrelationships at all are assumed among any of the items, where values < 0.90 are generally inadequate [28]. The SRMR is an absolute measure of fit capturing the standardized difference between the observed correlation and the predicted correlation. The threshold for acceptable model fit is considered when SRMR values ≤ 0.08 [28]. The results in Table B6 show that the unidimensional model fails to meet the criteria for model fit whereas the bifactor model either meets, or comes close to meeting, these criteria.

## **Table B5:** Model Outputs for the Unidimensional and Bifactor Models

|  | **Unidimensional Model** | | | **Bifactor Model** | | |
| --- | --- | --- | --- | --- | --- | --- |
|  | **Estimate** | **SE** | **P(>\|t\|)** | **Estimate** | **SE** | **P(>\|t\|)** |
| Standardized factor loadings (Common Factor -> SF-12) |  |  |  |  |  |  |
| General health | 0.702 | 0.004 | 0.000 | 0.712 | 0.004 | 0.000 |
| Limited in moderate activities | -0.896 | 0.003 | 0.000 | -0.877 | 0.003 | 0.000 |
| Limited in climbing stairs | -0.865 | 0.003 | 0.000 | -0.850 | 0.003 | 0.000 |
| Accomplished less (physical) | -0.933 | 0.002 | 0.000 | -0.912 | 0.003 | 0.000 |
| Limited work (physical) | -0.955 | 0.002 | 0.000 | -0.929 | 0.003 | 0.000 |
| Accomplished less (mental) | -0.814 | 0.005 | 0.000 | -0.779 | 0.006 | 0.000 |
| Limited work (mental) | -0.719 | 0.006 | 0.000 | -0.691 | 0.007 | 0.000 |
| Pain interfered with work | 0.844 | 0.003 | 0.000 | 0.850 | 0.003 | 0.000 |
| Felt calm and peaceful | 0.623 | 0.004 | 0.000 | 0.568 | 0.005 | 0.000 |
| Had a lot of energy | 0.729 | 0.004 | 0.000 | 0.724 | 0.004 | 0.000 |
| Felt downhearted and blue | -0.648 | 0.005 | 0.000 | -0.596 | 0.005 | 0.000 |
| Problems with social activities | -0.754 | 0.004 | 0.000 | -0.743 | 0.005 | 0.000 |
| Standardized factor loadings (Common Factor -> EQ-5D) |  |  |  |  |  |  |
| Mobility | 0.864 | 0.004 | 0.000 | 0.865 | 0.004 | 0.000 |
| Self-care | 0.809 | 0.007 | 0.000 | 0.812 | 0.007 | 0.000 |
| Usual activities | 0.916 | 0.003 | 0.000 | 0.923 | 0.003 | 0.000 |
| Pain | 0.811 | 0.003 | 0.000 | 0.817 | 0.003 | 0.000 |
| Anxiety | 0.762 | 0.005 | 0.000 | 0.816 | 0.005 | 0.000 |
| Standardized factor loadings (SF-12 Latent Factor -> SF-12) |  |  |  |  |  |  |
| General health |  |  |  | -0.045 | 0.007 | 0.000 |
| Limited in moderate activities |  |  |  | 0.306 | 0.006 | 0.000 |
| Limited in climbing stairs |  |  |  | 0.309 | 0.007 | 0.000 |
| Accomplished less (physical) |  |  |  | 0.252 | 0.007 | 0.000 |
| Limited work (physical) |  |  |  | 0.295 | 0.007 | 0.000 |
| Accomplished less (mental) |  |  |  | -0.443 | 0.009 | 0.000 |
| Limited work (mental) |  |  |  | -0.342 | 0.010 | 0.000 |
| Pain interfered with work |  |  |  | -0.111 | 0.006 | 0.000 |
| Felt calm and peaceful |  |  |  | 0.503 | 0.006 | 0.000 |
| Had a lot of energy |  |  |  | 0.235 | 0.006 | 0.000 |
| Felt downhearted and blue |  |  |  | -0.487 | 0.006 | 0.000 |
| Problems with social activities |  |  |  | -0.259 | 0.007 | 0.000 |
| Standardized factor loadings (EQ-5D Latent Factor -> EQ-5D) |  |  |  |  |  |  |
| Mobility |  |  |  | 0.266 | 0.010 | 0.000 |
| Self-care |  |  |  | 0.176 | 0.012 | 0.000 |
| Usual activities |  |  |  | 0.194 | 0.008 | 0.000 |
| Pain |  |  |  | 0.141 | 0.008 | 0.000 |
| Anxiety |  |  |  | -0.947 | 0.044 | 0.000 |
| Item category thresholds (SF-12) |  |  |  |  |  |  |
| General health \| 1 | -0.995 | 0.011 | 0.000 | -0.995 | 0.011 | 0.000 |
| General health \| 2 | 0.043 | 0.009 | 0.000 | 0.043 | 0.009 | 0.000 |
| General health \| 3 | 0.973 | 0.010 | 0.000 | 0.973 | 0.010 | 0.000 |
| General health \| 4 | 1.847 | 0.017 | 0.000 | 1.847 | 0.017 | 0.000 |
| Limited in moderate activities \| 1 | -1.342 | 0.012 | 0.000 | -1.342 | 0.012 | 0.000 |
| Limited in moderate activities \| 2 | -0.624 | 0.009 | 0.000 | -0.624 | 0.009 | 0.000 |
| Limited in climbing stairs \| 1 | -1.252 | 0.012 | 0.000 | -1.252 | 0.012 | 0.000 |
| Limited in climbing stairs \| 2 | -0.470 | 0.009 | 0.000 | -0.470 | 0.009 | 0.000 |
| Accomplished less (physical) \| 1 | -0.717 | 0.010 | 0.000 | -0.717 | 0.010 | 0.000 |
| Limited work (physical) \| 1 | -0.743 | 0.010 | 0.000 | -0.743 | 0.010 | 0.000 |

Table Bontinues on the next page

**Table B5: Model Outputs for the Unidimensional and Bifactor Models (Continued)**

|  | Unidimensional Model | | | Bifactor Model | | |
| --- | --- | --- | --- | --- | --- | --- |
|  | Estimate | SE | P(>\|t\|) | Estimate | SE | P(>\|t\|) |
| Item category thresholds (SF-12) |  |  |  |  |  |  |
| Accomplished less (mental) \| 1 | -0.876 | 0.010 | 0.000 | -0.876 | 0.010 | 0.000 |
| Limited work (mental) \| 1 | -0.873 | 0.010 | 0.000 | -0.873 | 0.010 | 0.000 |
| Pain interfered with work \| 1 | 0.090 | 0.009 | 0.000 | 0.090 | 0.009 | 0.000 |
| Pain interfered with work \| 2 | 0.778 | 0.010 | 0.000 | 0.778 | 0.010 | 0.000 |
| Pain interfered with work \| 3 | 1.239 | 0.012 | 0.000 | 1.239 | 0.012 | 0.000 |
| Pain interfered with work \| 4 | 1.898 | 0.018 | 0.000 | 1.898 | 0.018 | 0.000 |
| Felt calm and peaceful \| 1 | -1.163 | 0.011 | 0.000 | -1.163 | 0.011 | 0.000 |
| Felt calm and peaceful \| 2 | 0.077 | 0.009 | 0.000 | 0.077 | 0.009 | 0.000 |
| Felt calm and peaceful \| 3 | 0.579 | 0.009 | 0.000 | 0.579 | 0.009 | 0.000 |
| Felt calm and peaceful \| 4 | 1.272 | 0.012 | 0.000 | 1.272 | 0.012 | 0.000 |
| Felt calm and peaceful \| 5 | 1.945 | 0.018 | 0.000 | 1.945 | 0.019 | 0.000 |
| Had a lot of energy \| 1 | -1.276 | 0.012 | 0.000 | -1.276 | 0.012 | 0.000 |
| Had a lot of energy \| 2 | -0.030 | 0.009 | 0.000 | -0.030 | 0.009 | 0.001 |
| Had a lot of energy \| 3 | 0.483 | 0.009 | 0.000 | 0.483 | 0.009 | 0.000 |
| Had a lot of energy \| 4 | 1.158 | 0.011 | 0.000 | 1.158 | 0.011 | 0.000 |
| Had a lot of energy \| 5 | 1.753 | 0.016 | 0.000 | 1.753 | 0.016 | 0.000 |
| Felt downhearted and blue \| 1 | -2.158 | 0.022 | 0.000 | -2.158 | 0.022 | 0.000 |
| Felt downhearted and blue \| 2 | -1.620 | 0.015 | 0.000 | -1.620 | 0.015 | 0.000 |
| Felt downhearted and blue \| 3 | -1.229 | 0.012 | 0.000 | -1.229 | 0.012 | 0.000 |
| Felt downhearted and blue \| 4 | -0.519 | 0.009 | 0.000 | -0.519 | 0.009 | 0.000 |
| Felt downhearted and blue \| 5 | 0.430 | 0.009 | 0.000 | 0.430 | 0.009 | 0.000 |
| Problems with social activities \| 1 | -1.936 | 0.018 | 0.000 | -1.936 | 0.018 | 0.000 |
| Problems with social activities \| 2 | -1.478 | 0.013 | 0.000 | -1.478 | 0.013 | 0.000 |
| Problems with social activities \| 3 | -0.855 | 0.010 | 0.000 | -0.855 | 0.010 | 0.000 |
| Problems with social activities \| 4 | -0.368 | 0.009 | 0.000 | -0.368 | 0.009 | 0.000 |
| Item category thresholds (EQ-5D) |  |  |  |  |  |  |
| Mobility \| 1 | 0.865 | 0.010 | 0.000 | 0.865 | 0.010 | 0.000 |
| Mobility \| 2 | 2.594 | 0.035 | 0.000 | 2.594 | 0.035 | 0.000 |
| Self-care \| 1 | 1.654 | 0.015 | 0.000 | 1.654 | 0.015 | 0.000 |
| Self-care \| 2 | 2.455 | 0.030 | 0.000 | 2.455 | 0.030 | 0.000 |
| Usual activities \| 1 | 0.834 | 0.010 | 0.000 | 0.834 | 0.010 | 0.000 |
| Usual activities \| 2 | 1.997 | 0.019 | 0.000 | 1.997 | 0.019 | 0.000 |
| Pain \| 1 | 0.143 | 0.009 | 0.000 | 0.143 | 0.009 | 0.000 |
| Pain \| 2 | 1.740 | 0.016 | 0.000 | 1.740 | 0.016 | 0.000 |
| Anxiety \| 1 | 0.594 | 0.009 | 0.000 | 0.594 | 0.009 | 0.000 |
| Anxiety \| 2 | 1.948 | 0.018 | 0.000 | 1.948 | 0.019 | 0.000 |

## **Table B6:** Model Fit Statistics for the Unidimensional and Bifactor Models

|  | **Unidimensional model** | **Bifactor Model** |
| --- | --- | --- |
| **Root mean square error of approximation** | **0.120** | **0.094** |
| **Comparative fit index** | **0.907** | **0.951** |
| **Tucker-Lewis index** | **0.894** | **0.935** |
| **Standardized Root Mean Square Residual** | **0.115** | **0.081** |

**Face Validity and Predictive Performance**

Figure B3 presents a scatter plot of observed and predicted EQ-5D-3L values from both the unidimensional and bifactor models when applied to the external dataset (2000 MEPS). These data show similar trends to those in previous studies, especially the underprediction of values for people in full health, as well as those with higher values below full health [25]. The lack of predicted values equivalent to full health can be attributed to the use of the expected utility method. Although this may seem imperfect, it has been shown to produce better predictions when compared to alternative methods such as the Monte Carlo and Most-Likely Probability methods [24].

## **Figure B3:** Scatter Plot of Observed and Predicted EQ-5D-3L Values where the blue dots reflect predictions from the unidimensional LVM and the pink dots reflect predictions from the bifactor LVM. The diagonal line is included to show the point at which predicted and observed EQ-5D-3L would be equivalent


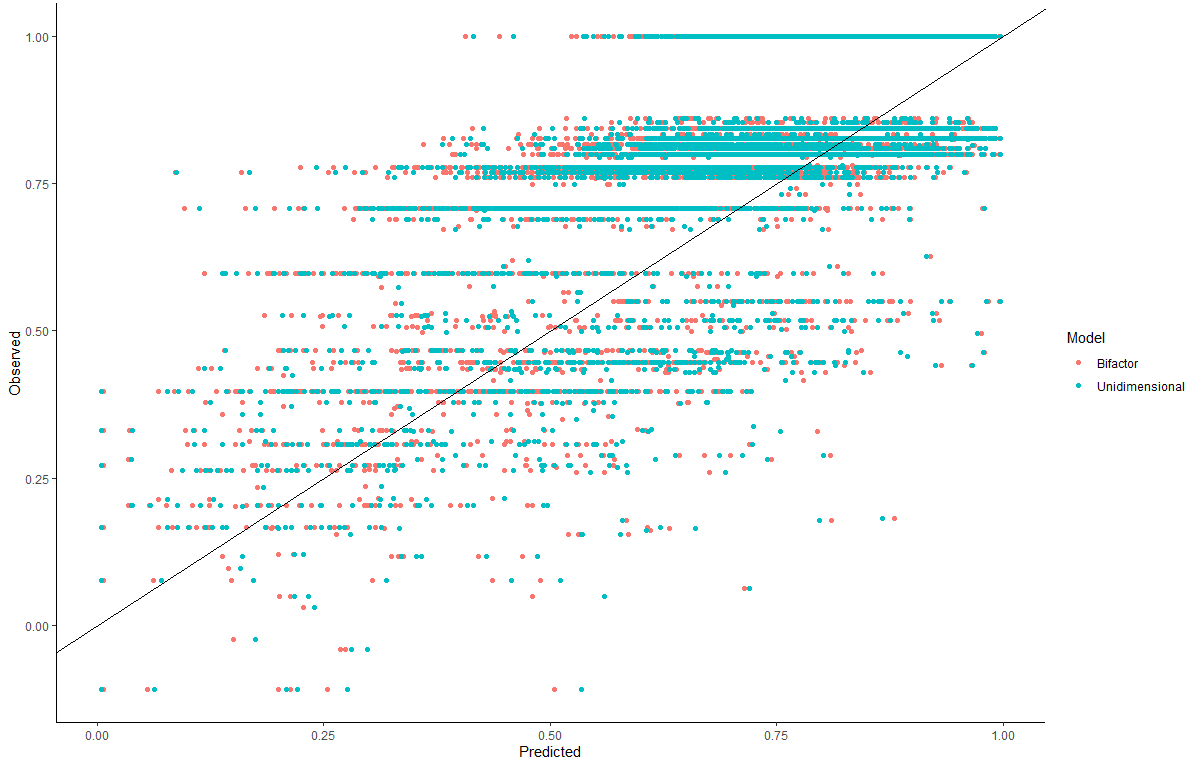


For predicted EQ-5D-3L scores from the unidimensional model in the external dataset, the mean absolute error (MAE) was 0.0919 (95% confidence intervals (CIs) from probabilistic predictions = 0.0917 – 0.0920) and the root mean squared error (RMSE) was 0.1215 (95% CIs from probabilistic predictions = 0.1213 – 0.1217). For predicted EQ-5D-3L scores from the bifactor model in the external dataset, the mean absolute error (MAE) was 0.0906 (95% CI = 0.0904 – 0.0908) and the root mean squared error (RMSE) was 0.1210 (95% CI = 0.1209 – 0.1212). The difference in performance between the two models is small, which is due to the same set of parameters (i.e. those related to the general factor) being used to inform predictions. However, the predictions from the bifactor model are theoretically more appropriate than the unidimensional model because it has controlled for instrument-specific variations when estimating parameters in the general factor. Tables B6 and B7 presents these metrics across a range of observed EQ-5D-3L values. The predictive performance of the mapping algorithm developed in this study is superior to that in a previous study mapping between the SF-12 Version 1 and the EQ-5D-3L with US population values [29]. Another study was found to have a model exhibiting superior predictive performance, albeit over specific segments of the EQ-5D-3L distribution [30]. However, performance in the study by Franks et al. was based on predictions in the same data used to derive the mapping algorithm and, as such, is less useful for understanding the generalizability of the predictions.

## **Table B7:** Mean absolute errors (95% confidence intervals)

| EQ-5D range | Unidimensional model | Bifactor model |
| --- | --- | --- |
| < 0 | 0.2635 (0.2627,0.2644) | 0.2534 (0.2527,0.2542) |
| 0 ≤ x < 0.25 | 0.1730 (0.1727,0.1733) | 0.1643 (0.1640,0.1646) |
| 0.25 ≤ x <0.5 | 0.1455 (0.1452,0.1458) | 0.1410 (0.1407,0.1413) |
| 0.5 ≤ x < 0.7 | 0.1769 (0.1766,0.1773) | 0.1839 (0.1836,0.1841) |
| 0.7 ≤ x < 0.8 | 0.1452 (0.1447,0.1457) | 0.1534 (0.1530,0.1539) |
| 0.8 ≤ x < 0.9 | 0.0760 (0.0759,0.0762) | 0.0763 (0.0762,0.0764) |
| 0.9 ≤ x < 1.0 | 0.0811 (0.0809,0.0813) | 0.0764 (0.0762,0.0766) |

## **Table B8:** Root mean squared errors (95% CI)

| EQ-5D range | Unidimensional model | Bifactor model |
| --- | --- | --- |
| < 0 | 0.3043 (0.3036,0.3050) | 0.2910 (0.2905,0.2917) |
| 0 ≤ x < 0.25 | 0.2320 (0.2317,0.2324) | 0.2243 (0.2240,0.2246) |
| 0.25 ≤ x <0.5 | 0.1803 (0.1799,0.1806) | 0.1773 (0.1770,0.1775) |
| 0.5 ≤ x < 0.7 | 0.2055 (0.2051,0.2059) | 0.2139 (0.2136,0.2142) |
| 0.7 ≤ x < 0.8 | 0.1789 (0.1784,0.1793) | 0.1885 (0.1881,0.1890) |
| 0.8 ≤ x < 0.9 | 0.0961 (0.0959,0.0963) | 0.0973 (0.0971,0.0974) |
| 0.9 ≤ x < 1.0 | 0.1063 (0.1061,0.1065) | 0.1003 (0.1000,0.1005) |

Figure B4 presents a histogram showing the distribution of prediction errors across a range of values (note the overlap between the distributions for the two methods). Within the external dataset, the percentage of absolute prediction errors below 0.05 was slightly higher for the bifactor model compared to the unidimensional model (36.4% versus 35.5%). The percentage of absolute prediction errors below 0.10 was also slightly higher for the bifactor model compared to the unidimensional model (66.4% versus 65.1%).For reference, values ranging between 0.03 and 0.08 have been purported to represent minimally important differences for the EQ-5D-3L with US population values [31–33].

## **Figure B4:** Histogram of absolute prediction errors


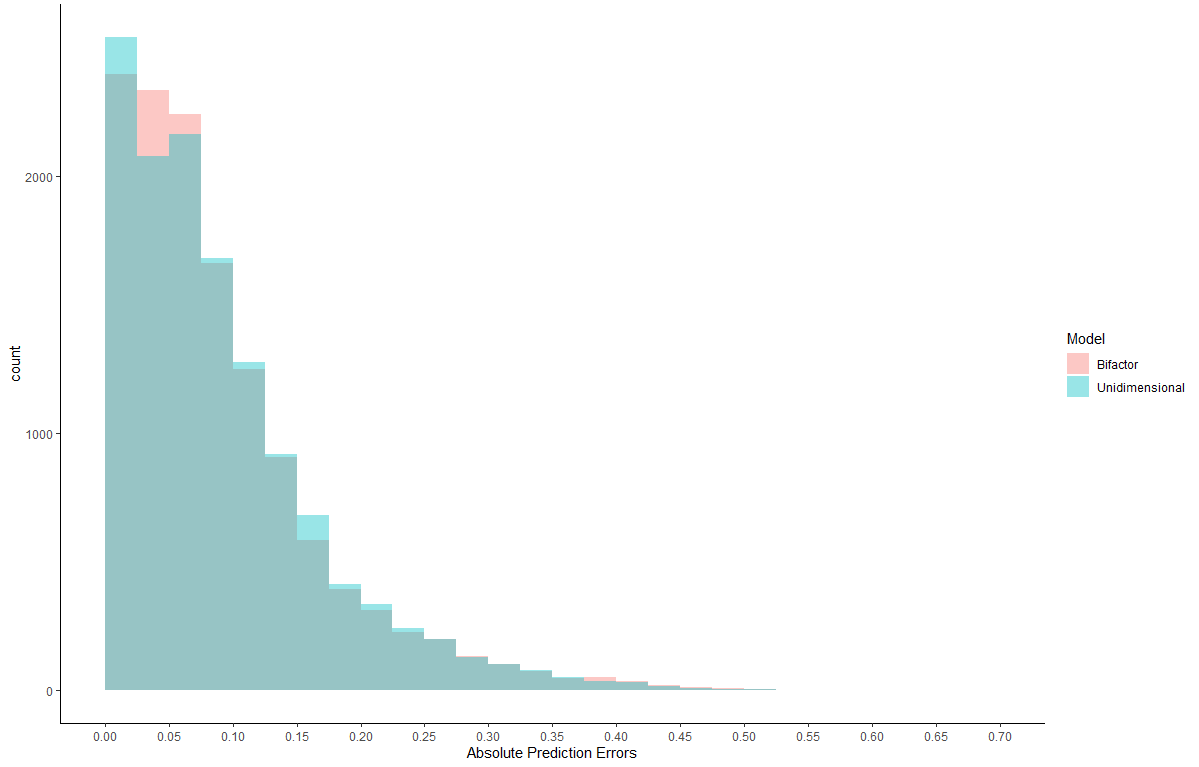


**Limitations of the Mapping Algorithms and Further Applications**

The mapping algorithms outlined in this section is not without limitations. First, the multiple imputation approach used to handle missing responses to the EQ-5D-3L and SF-12 Version 1 assumed that the data were missing at random. This assumption is only appropriate if the multivariate distributions underlying the observed and missing data are identical [18]. While multiple imputation techniques can also be utilized if the data are missing not at random, this approach requires additional assumptions and presents further challenges in terms of the estimation methods. Another limitation is the misalignment between the sample population used to derive the mapping algorithm (non-condition specific) and the target population for whom the algorithm has been developed to predict EQ-5D-3L responses (people with opioid use disorder). Despite this misalignment, the MEPS data is likely to represent the next best alternative, in lieu of a sample for estimating algorithms in the target population, given that it covers a diverse range of conditions and broad coverage of the health states captured by the two measures. While the primary purpose of the mapping algorithm developed in this section is to predict EQ-5D-3L responses using SF-12 Version 1 responses collected in NIDA trials, it may also prove to be useful in studies beyond the scope of this study. Previous research has indicated that mappings between generic HRQoL instruments are similar across different settings and medical conditions [34]. As such, the mapping algorithm may be useful for predicting EQ-5D-3L responses in US settings and for medical conditions outside the context of this paper. A final point to consider is the methodological question whether the analyzed HRQoL measures are appropriately modelled with models that assume quantitative latent variables. While this has been argued in the past [35, 36], these models assume that all responses in a scale can be reduced to a limited set of causes (one cause in the case of a unidimensional model) that proportionally affect the responses to all items. For instance, if the latent variable in the unidimensional model is increased by one standard deviation, the model predicts a change in all items proportional to their respective loading [37]. Qualitative variation, such as patterns that are mainly characterized by psychological impacts versus those that are characterized by physical impacts, are therefore not considered [38, 39]. While the high loadings in the present study suggest that a substantial amount of inter-individual variation is captured for both measures with either of the latent variable models, further methodological work is needed. An important area for future work would be to explore the consequences of conditions for specific groups or population segments, both empirically as well as in terms of theory building when using such an approach.

**Selection of the Preferred Mapping Algorithm**

The bifactor model was selected as the preferred mapping algorithm for the prediction of EQ-5D values because it met more of the criteria for model fit compared to the unidimensional model. The bifactor was also shown to have slightly better predictive performance when compared to the unidimensional model.

**‘Checklist of items to include when reporting a mapping study’** [40]

**Item 1**: Identify the report as a study mapping between outcome measures. State the source measure(s) and generic, preference-based target measure(s) used in the study

See title on page 6.

**Item 2**: Provide a structured abstract including, as applicable: objectives; methods, including data sources and their key characteristics, outcome measures used and estimation and validation strategies; results, including indicators of model performance; conclusions; and implications of key findings

Not applicable.

**Item 3**: Describe the rationale for the mapping study in the context of the broader evidence base

See section under the subheading ‘Study Rationale’ on page 6.

**Item 4**: Specify the research question with reference to the source and target measures used and the disease or population context of the study

See section under the subheading ‘Study Objective’ on page 6.

**Item 5**: Describe how the estimation sample was identified, why it was selected, the methods of recruitment and data collection, and its location(s) or setting(s)

See section under the subheading ‘Estimation Sample’ on page 6.

**Item 6**: If an external validation sample was used, the rationale for selection, the methods of recruitment and data collection, and its location(s) or setting(s) should be described

See section under the subheading ‘External Validation Sample’ on page 6.

**Item 7**: Describe the source and target measures and the methods by which they were applied in the mapping study

See section under the subheading ‘Source and Target Measures’ on page 7.

**Item 8**: Describe the methods used to assess the degree of conceptual overlap between the source and target measures

See section under the subheading ‘Conceptual Overlap Between Measures’ on page 7.

**Item 9**: State how much data were missing and how missing data were handled in the sample(s) used for the analyses

See section under the subheading ‘Exploratory Data Analysis’ on page 7.

**Item 10**: Describe and justify the statistical model(s) used to develop the mapping algorithm

See section under the subheading ‘Model Specification for Mapping Algorithms’ on page 11.

**Item 11**: Describe how predicted scores or utilities are estimated for each model specification

See section under the subheading ‘Prediction of EQ-5D-3L Responses’ on page 14.

**Item 12**: Describe and justify the methods used to validate the mapping algorithm

See section under the subheading ‘Validation of EQ-5D-3L Predictions and Measures of Model Performance’ on page 14.

**Item 13**: State and justify the measure(s) of model performance that determine the choice of the preferred model(s) and describe how these measures were estimated and applied

See section under the subheading ‘Validation of EQ-5D-3L Predictions and Measures of Model Performance’ on page 14.

**Item 14**: State the size of the estimation sample and any validation sample(s) used in the analyses (including both number of individuals and number of observations)

See section under the subheading ‘Sample Description’ on page 15.

**Item 15**: Describe the characteristics of individuals in the sample(s) (or refer back to previous publications giving such information). Provide summary scores for source and target measures, and summarize results of analyses used to assess overlap between the source and target measures

See the section under the subheading ‘Sample Description’ on page 15 and the section under the subheading ‘Conceptual Overlap Between Measures’ on page 7.

**Item 16**: State which model(s) is (are) preferred and justify why this (these) model(s) was (were) chosen

See the section under the subheading ‘Model Outputs’ on page 15.

**Item 17**: Provide all model coefficients and standard errors for the selected model(s). Provide clear guidance on how a user can calculate utility scores based on the outputs of the selected model(s)

See the section under the subheading ‘Model Outputs’ on page 15.

**Item 18**: Report information that enables users to estimate standard errors around mean utility predictions and individual-level variability

See the section under the subheading ‘Model Outputs’ on page 15.

**Item 19**: Present results of model performance, such as measures of prediction accuracy and fit statistics for the selected model(s) in a table or in the text. Provide an assessment of face validity of the selected model(s)

See the section under the subheading ‘Face Validity and Predictive Performance’ on page 18.

**Item 20**: Report details of previously published studies developing mapping algorithms between the same source and target measures and describe differences between the algorithms, in terms of model performance, predictions and coefficients, if applicable

See the section under the subheading ‘Face Validity and Predictive Performance’ on page 18.

**Item 21**: Outline the potential limitations of the mapping algorithm

See the section under the subheading ‘Limitations of the Mapping Algorithms and Further Applications’ on page 20.

**Item 22**: Outline the clinical and research settings in which the mapping algorithm could be used

See the section under the subheading ‘Limitations of the Mapping Algorithms and Further Applications’ on page 20.

**Item 23**: Describe the source(s) of funding and non-monetary support for the study, and the role of the funder(s) in its design, conduct and report. Report any conflicts of interest surrounding the roles of authors and funders

See declarations of interest in the main text.

# APPENDIX C: MAPPING BETWEEN SF-12 VERSION 2 AND EQ-5D-3L

**Title**

Mapping responses to the SF-12 Version 2 onto responses to the EQ-5D-3L using survey data collected in a sample of the United States (U.S.) population.

**Study Rationale**

The rationale for developing mapping algorithms in this paper is to support the comprehensive use of health-related quality of life (HRQoL) evidence from the National Institute on Drug Abuse (NIDA) Data Share initiative to represent the health burden associated with health states typically found in opioid use disorder (OUD) models in the published literature. This approach avoids disregarding potentially relevant evidence, simply because it has not been produced using a specific HRQoL measure.

**Study Objective**

The purpose of this analysis is to develop a mapping algorithm linking the SF-12 Version 2 (source measure) and the EQ-5D-3L (target measure) using data from the 2003 Medical Expenditure Panel Survey (MEPS), which is a nationally representative survey of the non-institutionalized U.S. population [24]. The development of this algorithm is intended to facilitate the prediction of EQ-5D-3L responses using SF-12 Version 2 data collected in trials identified through the NIDA Data Share initiative and the estimation of health index values reflecting US population-based preference weights [7].

**Estimation Sample**

For SF-12 Version 2 and EQ-5D-3L, data were obtained from the Household Component of the 2003 MEPS. This data has been used in previous mapping studies linking these measures [24]. The 2003 MEPS data provides a nationally representative indication of health care use, expenditures, sources of payment, and health insurance coverage for the U.S. civilian non-institutionalized population. In 2003, the Household Component of MEPS also provided estimates of respondents' demographic and socio-economic characteristics, as well as their self-reported health status measured through the SF-12 Version 2 and the EQ-5D-3L [10]. The sampling frame for the MEPS HC was drawn from respondents to National Health Interview Survey, which was based on a stratified multistage sample design [11], and included responses from individuals located throughout the United States. The rationale for its use as the estimation sample was twofold: (i) the data are publicly available online (from <https://www.meps.ahrq.gov/>), and (ii) it provides a nationally representative survey of the non-institutionalized U.S. population.

**External Validation Sample**

To test the external validity of the mapping algorithm developed, data were obtained from the National Health Measurement Study (NHMS). These data were collected between 2005 and 2006 in a sample of 3,844 people, aged between 35 and 89 years old, in the continental United States [41]. Respondents were contacted using random digit dialed telephone survey methods and asked to complete various questionnaires including the EQ-5D-3L and the SF-36 Version 2, from which specific items corresponding to the SF-12 Version 2 were selected for analysis. The MEPS data was preferred for the main analysis over the NHMS data due to it having a much larger sample size.

**Source and Target Measures**

The EQ-5D-3L was designated as the target measure for valuing HRQoL in this study following previous research indicating it to be the preferable generic, indirect utility measure [12]. The EQ-5D-3L has two components [13]: (i) a descriptive system comprised of five dimensions (mobility, self-care, usual activities, pain/discomfort, and anxiety/depression), each of which has 3 levels (no problems, some problems, and extreme problems), and (ii) a value set reflecting preferences for the different health states within the descriptive system among a sample of the jurisdiction of interest. The source measure of HRQoL in the mapping exercises was the SF-12 Version 2, which is a self-reported outcome measure assessing the impact of health on an individual’s everyday life [42]. The SF-12 Version 2 is composed of twelve questions relating to eight health domains (physical functioning, role-physical, bodily pain, general health, vitality, social functioning, role-emotional, and mental health).

**Conceptual Overlap Between Measures**

No exploratory analysis to demonstrate conceptual overlap between the EQ-5D-3L and SF-12 (Version 2) measures was considered necessary given that previous research has already provided empirical support for this overlap to justify mapping between them. A study by Cheak-Zomara and colleagues tested the convergent validity of the EQ-5D-3L and SF-12 Version 2 using the 2003 MEPS data and found that the measures were overlapping in the measurement of mental and physical health constructs [43].

**Exploratory Data Analysis**

Of the 20,742 individuals providing any response to both the SF-12 Version 2 and the EQ-5D, 19,678 individuals provided complete responses to all items, which equates to 5.1% of respondents with missing data. Table C1 provides a breakdown of the level of missing data for the two instruments among the people with more than one item without a response. Table C2 shows EQ-5D responses across differing levels of missingness on the SF-12 and Table C3 shows SF-12 responses across differing levels of missingness on the EQ-5D. These data do not exhibit clear trends as per the exploratory analyses conducted in Appendix B.

Observations were dropped if they were missing any more than 50% of items for either of the measurements (N=52). A hypothesis test (Little, 1988) was performed on the remaining data, which showed that the data are not missing completely at random, i.e. p-value for the hypothesis that the data are Missing Completely At Random (MCAR) was 0. As such, the data were assumed to be Missing at Random (MAR) and multiple imputation was used to deal with the missing data (Van Buuren & Groothuis-Oudshoorn, 2011). This approach predicts a response for each item on the SF-12 Version 2 and EQ-5D-3L with missing variables conditional on the response to all other items available. No auxiliary variables were included. Five sets of imputed data were produced via multivariate imputation with chained equations. The mapping models (described in the next section) were then fitted to each of the imputed datasets and the outputs were then combined via pooling methods.

## **Table C1:** Missing data patterns in sample responding to both the EQ-5D-3L and the SF-12 Version 2

|  | | **Number of EQ-5D items with missing data** | | | | |  | **Row totals** |
| --- | --- | --- | --- | --- | --- | --- | --- | --- |
|  |  | **0** | **1** | **2** | **3** | **4** | **5** |  |
| **Number of SF-12 items with missing data** | **0** | 19,678 | 174 | 21 | 13 | 10 | 12 | 19,908 |
|  | **1** | 584 | 30 | 8 | 3 | 2 | 1 | 628 |
|  | **2** | 94 | 18 | 4 | 1 | 1 | 2 | 120 |
|  | **3** | 20 | 2 | 1 | 0 | 1 | 0 | 24 |
|  | **4** | 30 | 5 | 2 | 2 | 0 | 9 | 48 |
|  | **5** | 6 | 2 | 1 | 0 | 0 | 6 | 15 |
|  | **6** | 2 | 1 | 1 | 0 | 0 | 1 | 5 |
|  | **7** | 6 | 3 | 2 | 0 | 0 | 2 | 13 |
|  | **8** | 6 | 0 | 0 | 0 | 2 | 2 | 10 |
|  | **9** | 0 | 0 | 1 | 2 | 0 | 2 | 5 |
|  | **10** | 1 | 0 | 0 | 0 | 1 | 1 | 3 |
|  | **11** | 1 | 0 | 0 | 0 | 0 | 3 | 4 |
|  | **12** | 0 | 0 | 0 | 0 | 0 | 13,432 | 13,432 |
| **Column totals** | | 20,428 | 235 | 41 | 21 | 17 | 13,473 | **20,742** |

## **Table C2:** EQ-5D-3L responses by levels of missingness on the SF-12 Version 2

|  | Number of SF-12 items with missing data | | | | | | | | | | | |  |
| --- | --- | --- | --- | --- | --- | --- | --- | --- | --- | --- | --- | --- | --- |
|  | 0 | 1 | 2 | 3 | 4 | 5 | 6 | 7 | 8 | 9 | 10 | 11 | 12 |
| Mobility = No problems | 80.6% | 71.9% | 72.3% | 80.0% | 70.0% | 66.7% | 50.0% | 66.7% | 66.7% | 0.0% | 100.0% | 100.0% | 0.0% |
| Mobility = Some problems | 18.9% | 26.5% | 26.6% | 20.0% | 30.0% | 33.3% | 0.0% | 33.3% | 33.3% | 0.0% | 0.0% | 0.0% | 0.0% |
| Mobility = Confined to bed | 0.5% | 1.5% | 1.1% | 0.0% | 0.0% | 0.0% | 50.0% | 0.0% | 0.0% | 0.0% | 0.0% | 0.0% | 0.0% |
| Self care = No problems | 94.7% | 89.9% | 88.3% | 75.0% | 96.7% | 100.0% | 50.0% | 100.0% | 83.3% | 0.0% | 100.0% | 100.0% | 0.0% |
| Self care = Some problems | 4.6% | 8.2% | 9.6% | 10.0% | 3.3% | 0.0% | 0.0% | 0.0% | 16.7% | 0.0% | 0.0% | 0.0% | 0.0% |
| Self care = Unable to wash/dress | 0.7% | 1.9% | 2.1% | 15.0% | 0.0% | 0.0% | 50.0% | 0.0% | 0.0% | 0.0% | 0.0% | 0.0% | 0.0% |
| Usual activities = No problems | 79.3% | 68.0% | 69.1% | 65.0% | 70.0% | 100.0% | 50.0% | 83.3% | 50.0% | 0.0% | 0.0% | 100.0% | 0.0% |
| Usual activities = Some problems | 18.4% | 27.2% | 26.6% | 30.0% | 26.7% | 0.0% | 0.0% | 16.7% | 50.0% | 0.0% | 100.0% | 0.0% | 0.0% |
| Usual activities = Unable to perform | 2.4% | 4.8% | 4.3% | 5.0% | 3.3% | 0.0% | 50.0% | 0.0% | 0.0% | 0.0% | 0.0% | 0.0% | 0.0% |
| Pain = None | 57.5% | 49.5% | 53.2% | 65.0% | 43.3% | 50.0% | 50.0% | 66.7% | 66.7% | 0.0% | 0.0% | 100.0% | 0.0% |
| Pain = Moderate | 38.0% | 41.1% | 41.5% | 20.0% | 53.3% | 50.0% | 50.0% | 16.7% | 33.3% | 0.0% | 100.0% | 0.0% | 0.0% |
| Pain = Extreme | 4.5% | 5.3% | 5.3% | 15.0% | 3.3% | 0.0% | 0.0% | 16.7% | 0.0% | 0.0% | 0.0% | 0.0% | 0.0% |
| Anxiety = None | 71.9% | 63.4% | 66.0% | 70.0% | 56.7% | 50.0% | 50.0% | 100.0% | 66.7% | 0.0% | 0.0% | 100.0% | 0.0% |
| Anxiety = Moderate | 25.2% | 31.7% | 30.9% | 25.0% | 43.3% | 50.0% | 50.0% | 0.0% | 33.3% | 0.0% | 0.0% | 0.0% | 0.0% |
| Anxiety = Extreme | 2.9% | 5.0% | 3.2% | 5.0% | 0.0% | 0.0% | 0.0% | 0.0% | 0.0% | 0.0% | 100.0% | 0.0% | 0.0% |

## **Table C3:** SF-12 Version 2 responses by levels of missingness on the EQ-5D-3L

|  | Number of EQ-5D-3L items with missing data | | | | | |
| --- | --- | --- | --- | --- | --- | --- |
|  | 0 | 1 | 2 | 3 | 4 | 5 |
| General Health = Excellent | 18.6% | 11.5% | 19.0% | 30.8% | 20.0% | 41.7% |
| General Health = Very good | 33.7% | 19.0% | 23.8% | 7.7% | 10.0% | 33.3% |
| General Health = Good | 31.2% | 39.7% | 33.3% | 46.2% | 40.0% | 25.0% |
| General Health = Fair | 12.8% | 20.1% | 14.3% | 0.0% | 30.0% | 0.0% |
| General Health = Poor | 3.7% | 9.8% | 9.5% | 15.4% | 0.0% | 0.0% |
| Accomplish less (mental) = All of the time | 2.9% | 6.3% | 4.8% | 15.4% | 0.0% | 16.7% |
| Accomplish less (mental) = Most of the time | 4.6% | 8.0% | 9.5% | 0.0% | 0.0% | 0.0% |
| Accomplish less (mental) = Some of the time | 12.7% | 24.1% | 14.3% | 30.8% | 10.0% | 0.0% |
| Accomplish less (mental) = A little of the time | 17.4% | 21.3% | 23.8% | 15.4% | 20.0% | 16.7% |
| Accomplish less (mental) = None of the time | 62.4% | 40.2% | 47.6% | 38.5% | 70.0% | 66.7% |
| Accomplish less (physical) = All of the time | 5.0% | 13.8% | 19.0% | 7.7% | 0.0% | 8.3% |
| Accomplish less (physical) = Most of the time | 7.1% | 12.6% | 4.8% | 0.0% | 10.0% | 8.3% |
| Accomplish less (physical) = Some of the time | 15.4% | 23.6% | 23.8% | 38.5% | 20.0% | 8.3% |
| Accomplish less (physical) = A little of the time | 18.6% | 16.7% | 23.8% | 7.7% | 10.0% | 8.3% |
| Accomplish less (physical) = None of the time | 53.9% | 33.3% | 28.6% | 46.2% | 60.0% | 8.3% |
| Felt calm/peaceful = All the time | 15.0% | 13.2% | 19.0% | 15.4% | 30.0% | 41.7% |
| Felt calm/peaceful = Most of the time | 47.9% | 37.9% | 47.6% | 15.4% | 20.0% | 41.7% |
| Felt calm/peaceful = Some of the time | 24.0% | 29.9% | 19.0% | 7.7% | 20.0% | 8.3% |
| Felt calm/peaceful = A little of the time | 9.1% | 11.5% | 14.3% | 30.8% | 20.0% | 0.0% |
| Felt calm/peaceful = None of the time | 4.0% | 7.5% | 0.0% | 30.8% | 10.0% | 8.3% |
| Limited moderate activities = Yes, limited a lot | 9.7% | 28.7% | 14.3% | 30.8% | 10.0% | 16.7% |
| Limited moderate activities = Yes, limited a little | 16.2% | 21.3% | 28.6% | 30.8% | 40.0% | 16.7% |
| Limited moderate activities = No, not limited at all | 74.1% | 50.0% | 57.1% | 38.5% | 50.0% | 66.7% |
| Felt downhearted/blue = All the time | 2.0% | 6.3% | 4.8% | 7.7% | 10.0% | 0.0% |
| Felt downhearted/blue = Most of the time | 5.4% | 6.3% | 0.0% | 7.7% | 0.0% | 8.3% |
| Felt downhearted/blue = Some of the time | 20.4% | 28.7% | 33.3% | 7.7% | 10.0% | 16.7% |
| Felt downhearted/blue = A little of the time | 30.8% | 24.7% | 19.0% | 23.1% | 10.0% | 16.7% |
| Felt downhearted/blue = None of the time | 41.4% | 33.9% | 42.9% | 53.8% | 70.0% | 58.3% |
| Limited work (mental) = All of the time | 2.6% | 6.9% | 0.0% | 7.7% | 0.0% | 8.3% |
| Limited work (mental) = Most of the time | 3.9% | 9.2% | 9.5% | 15.4% | 0.0% | 0.0% |

**Table C3**: SF-12 Version 2 responses by differing levels of missingness on the EQ-5D-3L (Continued)

|  | Number of EQ-5D-3L items with missing data | | | | | |
| --- | --- | --- | --- | --- | --- | --- |
|  | 0 | 1 | 2 | 3 | 4 | 5 |
| Limited work (mental) = Some of the time | 11.2% | 22.4% | 19.0% | 23.1% | 10.0% | 8.3% |
| Limited work (mental) = A little of the time | 17.8% | 17.2% | 9.5% | 7.7% | 20.0% | 8.3% |
| Limited work (mental) = None of the time | 64.4% | 44.3% | 61.9% | 46.2% | 70.0% | 75.0% |
| Limited work (physical) = All of the time | 4.8% | 15.5% | 14.3% | 7.7% | 10.0% | 16.7% |
| Limited work (physical) = Most of the time | 5.7% | 17.8% | 4.8% | 7.7% | 0.0% | 8.3% |
| Limited work (physical) = Some of the time | 12.8% | 19.5% | 19.0% | 30.8% | 30.0% | 8.3% |
| Limited work (physical) = A little of the time | 15.6% | 12.1% | 14.3% | 7.7% | 10.0% | 8.3% |
| Limited work (physical) = None of the time | 61.1% | 35.1% | 47.6% | 46.2% | 50.0% | 58.3% |
| Lots of energy = All the time | 13.4% | 14.4% | 19.0% | 15.4% | 20.0% | 8.3% |
| Lots of energy = Most of the time | 44.3% | 34.5% | 38.1% | 15.4% | 30.0% | 58.3% |
| Lots of energy = Some of the time | 26.5% | 27.0% | 28.6% | 23.1% | 40.0% | 25.0% |
| Lots of energy = A little of the time | 10.7% | 14.4% | 0.0% | 15.4% | 10.0% | 0.0% |
| Lots of energy = None of the time | 5.1% | 9.8% | 14.3% | 30.8% | 0.0% | 8.3% |
| Health stopped social activities = All of the time | 2.9% | 11.5% | 4.8% | 7.7% | 0.0% | 8.3% |
| Health stopped social activities = Most of the time | 5.2% | 9.2% | 9.5% | 0.0% | 0.0% | 16.7% |
| Health stopped social activities = Some of the time | 12.9% | 21.3% | 19.0% | 23.1% | 20.0% | 8.3% |
| Health stopped social activities = A little of the time | 16.4% | 16.1% | 9.5% | 7.7% | 10.0% | 0.0% |
| Health stopped social activities = None of the time | 62.6% | 42.0% | 57.1% | 61.5% | 70.0% | 66.7% |
| Pain limits work = Not at all | 54.7% | 33.9% | 38.1% | 53.8% | 70.0% | 58.3% |
| Pain limits work = A little bit | 23.8% | 27.6% | 42.9% | 30.8% | 10.0% | 0.0% |
| Pain limits work = Moderately | 10.1% | 13.8% | 4.8% | 0.0% | 20.0% | 8.3% |
| Pain limits work = Quite a bit | 7.9% | 16.1% | 9.5% | 15.4% | 0.0% | 8.3% |
| Pain limits work = Extremely | 3.6% | 8.6% | 4.8% | 0.0% | 0.0% | 25.0% |
| Health limits climbing stairs = Yes, limited a lot | 11.3% | 28.7% | 19.0% | 7.7% | 0.0% | 0.0% |
| Health limits climbing stairs = Yes, limited a little | 19.8% | 29.3% | 28.6% | 53.8% | 50.0% | 25.0% |
| Health limits climbing stairs = No, not limited at all | 68.9% | 42.0% | 52.4% | 38.5% | 50.0% | 75.0% |

**Model Specification for Mapping Algorithms**

The model specifications set out in Appendix B are applicable for the development of mapping algorithms linking the EQ-5D-3L and the SF-12 Version 2, at least in terms of the relationship between the different items. Aside from two items being reworded, the updated version of the SF-12 differs from the original in two ways [44]. First, the number of response categories were increased for items captured within the role-physical and role-emotional domains from two to five. Second, the number of response categories were decreased for items captured within the vitality and mental health domains from six to five. As such, the number of item category thresholds included in the model outputs from the mapping algorithms for the SF-12 Version 2 differ from those in the models in Appendix B. However, the graphical representations set out in Figures B1 and B2 are applicable for the development of mapping algorithms linking the EQ-5D-3L and the SF-12 Version 2. As with the analyses in Appendix B, both unidimensional and bifactor models were fitted to the 2003 MEPS data using the Lavaan package in R [21].

**Prediction of EQ-5D-3L Responses**

The same methods from Appendix B are applied for the prediction of EQ-5D-3L responses mapped from the SF-12 Version 2.

**Validation of EQ-5D-3L Predictions and Measures of Model Performance**

The same methods from Appendix B are applied to validate the EQ-5D-3L predictions and assess the model performance of the mapping algorithms.

**Results**

**Sample Description**

Twenty thousand six hundred and eighty four individuals from the 2003 MEPS study provided the minimum number of items required for inclusion in the sample for analysis (i.e. 50% or more of the items per instrument). Out of this sample, nineteen thousand six hundred and seventy eight provided responses for all the EQ-5D-3L and SF-12 Version 1 items. Missing data were imputed using multivariate imputation by chained equations techniques [18]. The validation sample (2000 MEPS study) had twelve thousand nine hundred and ninety eight individuals providing complete responses for all the EQ-5D-3L and SF-12 Version 1 items. Table C4 provides descriptive statistics for the final data used in the mapping analysis and for the data used for the validation of the mapping algorithms.

## **Table C4:** Sample Demographic Characteristics

|  | 2003 MEPS | NHMS |
| --- | --- | --- |
| Age (years) |  |  |
| -Mean | 45 | 60 |
| -Lower quartile | 31 | 48 |
| -Median | 44 | 60 |
| -Upper quartile | 57 | 71 |
| -Minimum | 18 | 35 |
| -Maximum | 85 | 89 |
| Sex |  |  |
| -Female (%) | 55% | 57% |
| US Census Region |  |  |
| -Northeast (%) | 15% | 15% |
| -Midwest (%) | 20% | 27% |
| -South (%) | 40% | 47% |
| -West (%) | 26% | 10% |
| -Not stated (%) | <1% | 0% |
| EQ-5D |  |  |
| -Mean | 0.859 | 0.840 |
| -Lower quartile | 0.800 | 0.800 |
| -Median | 0.844 | 0.827 |
| -Upper quartile | 1.000 | 1.000 |
| -Minimum | -0.109 | -0.109 |
| -Maximum | 1.000 | 1.000 |

MEPS = Medical Expenditure Panel Survey

**Model Outputs**

Table C5 shows the outputs from the unidimensional and bifactor models. A pooled variance covariance matrix was produced to capture the correlations between each of the model parameters when characterizing the sample uncertainty associated with the parameter estimates in the mapping algorithm. As in Appendix B, the following tests of model fit were performed: chi-squared test, the root mean square error of approximation (RMSEA), the comparative fit index (CFI), and the standardized root mean square residual (SRMR). The chi-squared test indicated a good model fit for both the unidimensional and bifactor models (p<0.05). The remaining test results are shown in Table C6. The RMSEA is a parsimony-adjusted index, where values >0.10 are regarded as being indicative of poor fit between the hypothesized model and the observed data. The CFI is a relative measure that compares the fit of a target model to the fit of an independent (or null) model, where values ≥ 0.95 have been proposed as representing an accepTable Eit [28]. The Tucker-Lewis index (TLI) compares the proposed factor model to a model in which no interrelationships at all are assumed among any of the items, where values < 0.90 are generally inadequate [28]. The SRMR is an absolute measure of fit capturing the standardized difference between the observed correlation and the predicted correlation. The threshold for acceptable model fit is considered when SRMR values ≤ 0.08 [28]. The results in Table C6 show that the unidimensional model fails to meet the criteria for model fit whereas the bifactor model either meets, or comes close to meeting, this criteria.

## **Table C5:** Model Outputs for the Unidimensional and Bifactor Models

|  | **Unidimensional Model** | | | **Bifactor Model** | | |
| --- | --- | --- | --- | --- | --- | --- |
|  | **Estimate** | **SE** | **P(>\|t\|)** | **Estimate** | **SE** | **P(>\|t\|)** |
| Standardized factor loadings (Common Factor -> SF-12) |  |  |  |  |  |  |
| General health | 0.718 | 0.003 | 0.000 | 0.727 | 0.003 | 0.000 |
| Limited in moderate activities | -0.909 | 0.002 | 0.000 | -0.888 | 0.003 | 0.000 |
| Limited in climbing stairs | -0.884 | 0.002 | 0.000 | -0.867 | 0.003 | 0.000 |
| Accomplished less (physical) | -0.891 | 0.002 | 0.000 | -0.888 | 0.002 | 0.000 |
| Limited work (physical) | -0.935 | 0.001 | 0.000 | -0.925 | 0.002 | 0.000 |
| Accomplished less (mental) | -0.830 | 0.003 | 0.000 | -0.800 | 0.004 | 0.000 |
| Limited work (mental) | -0.797 | 0.004 | 0.000 | -0.785 | 0.004 | 0.000 |
| Pain interfered with work | 0.820 | 0.003 | 0.000 | 0.826 | 0.003 | 0.000 |
| Felt calm and peaceful | 0.575 | 0.005 | 0.000 | 0.535 | 0.006 | 0.000 |
| Had a lot of energy | 0.699 | 0.004 | 0.000 | 0.699 | 0.004 | 0.000 |
| Felt downhearted and blue | -0.724 | 0.004 | 0.000 | -0.670 | 0.005 | 0.000 |
| Problems with social activities | -0.807 | 0.003 | 0.000 | -0.792 | 0.004 | 0.000 |
| Standardized factor loadings (Common Factor -> EQ-5D) |  |  |  |  |  |  |
| Mobility | 0.863 | 0.004 | 0.000 | 0.854 | 0.004 | 0.000 |
| Self-care | 0.787 | 0.006 | 0.000 | 0.781 | 0.007 | 0.000 |
| Usual activities | 0.908 | 0.003 | 0.000 | 0.904 | 0.003 | 0.000 |
| Pain | 0.815 | 0.003 | 0.000 | 0.815 | 0.003 | 0.000 |
| Anxiety | 0.788 | 0.004 | 0.000 | 0.831 | 0.004 | 0.000 |
| Standardized factor loadings (SF-12 Latent Factor -> SF-12) |  |  |  |  |  |  |
| General health |  |  |  | -0.032 | 0.006 | 0.000 |
| Limited in moderate activities |  |  |  | 0.318 | 0.006 | 0.000 |
| Limited in climbing stairs |  |  |  | 0.307 | 0.007 | 0.000 |
| Accomplished less (physical) |  |  |  | 0.171 | 0.006 | 0.000 |
| Limited work (physical) |  |  |  | 0.213 | 0.006 | 0.000 |
| Accomplished less (mental) |  |  |  | -0.377 | 0.007 | 0.000 |
| Limited work (mental) |  |  |  | -0.258 | 0.007 | 0.000 |
| Pain interfered with work |  |  |  | -0.109 | 0.006 | 0.000 |
| Felt calm and peaceful |  |  |  | 0.413 | 0.007 | 0.000 |
| Had a lot of energy |  |  |  | 0.175 | 0.006 | 0.000 |
| Felt downhearted and blue |  |  |  | -0.510 | 0.006 | 0.000 |
| Problems with social activities |  |  |  | -0.293 | 0.006 | 0.000 |
| Standardized factor loadings (EQ-5D Latent Factor -> EQ-5D) |  |  |  |  |  |  |
| Mobility |  |  |  | 0.342 | 0.009 | 0.000 |
| Self-care |  |  |  | 0.270 | 0.012 | 0.000 |
| Usual activities |  |  |  | 0.242 | 0.007 | 0.000 |
| Pain |  |  |  | 0.179 | 0.008 | 0.000 |
| Anxiety |  |  |  | -0.595 | 0.023 | 0.000 |
| Item category thresholds (SF-12) |  |  |  |  |  |  |
| General health \| 1 | -0.901 | 0.010 | 0.000 | -0.901 | 0.010 | 0.000 |
| General health \| 2 | 0.038 | 0.009 | 0.000 | 0.038 | 0.009 | 0.000 |
| General health \| 3 | 0.950 | 0.010 | 0.000 | 0.950 | 0.010 | 0.000 |
| General health \| 4 | 1.762 | 0.016 | 0.000 | 1.762 | 0.016 | 0.000 |
| Limited in moderate activities \| 1 | -1.266 | 0.012 | 0.000 | -1.266 | 0.012 | 0.000 |
| Limited in moderate activities \| 2 | -0.621 | 0.009 | 0.000 | -0.621 | 0.009 | 0.000 |
| Limited in climbing stairs \| 1 | -1.188 | 0.011 | 0.000 | -1.188 | 0.011 | 0.000 |
| Limited in climbing stairs \| 2 | -0.473 | 0.009 | 0.000 | -0.473 | 0.009 | 0.000 |
| Accomplished less (physical) \| 1 | -1.617 | 0.014 | 0.000 | -1.617 | 0.014 | 0.000 |
| Accomplished less (physical) \| 2 | -1.143 | 0.011 | 0.000 | -1.143 | 0.011 | 0.000 |

Table Bontinues on the next page

**Table C5:** Model Outputs for the Unidimensional and Bifactor Models (Continued)

|  | Unidimensional Model | | | Bifactor Model | | |
| --- | --- | --- | --- | --- | --- | --- |
|  | Estimate | SE | P(>\|t\|) | Estimate | SE | P(>\|t\|) |
| Item category thresholds (SF-12) |  |  |  |  |  |  |
| Accomplished less (physical) \| 3 | -0.574 | 0.009 | 0.000 | -0.574 | 0.009 | 0.000 |
| Accomplished less (physical) \| 4 | -0.080 | 0.009 | 0.000 | -0.080 | 0.009 | 0.000 |
| Limited work (physical) \| 1 | -1.626 | 0.015 | 0.000 | -1.626 | 0.015 | 0.000 |
| Limited work (physical) \| 2 | -1.216 | 0.012 | 0.000 | -1.216 | 0.012 | 0.000 |
| Limited work (physical) \| 3 | -0.697 | 0.010 | 0.000 | -0.697 | 0.010 | 0.000 |
| Limited work (physical) \| 4 | -0.260 | 0.009 | 0.000 | -0.260 | 0.009 | 0.000 |
| Accomplished less (mental) \| 1 | -1.876 | 0.017 | 0.000 | -1.876 | 0.017 | 0.000 |
| Accomplished less (mental) \| 2 | -1.413 | 0.013 | 0.000 | -1.413 | 0.013 | 0.000 |
| Accomplished less (mental) \| 3 | -0.810 | 0.010 | 0.000 | -0.810 | 0.010 | 0.000 |
| Accomplished less (mental) \| 4 | -0.299 | 0.009 | 0.000 | -0.299 | 0.009 | 0.000 |
| Limited work (mental) \| 1 | -1.907 | 0.018 | 0.000 | -1.907 | 0.018 | 0.000 |
| Limited work (mental) \| 2 | -1.483 | 0.013 | 0.000 | -1.483 | 0.013 | 0.000 |
| Limited work (mental) \| 3 | -0.897 | 0.010 | 0.000 | -0.897 | 0.010 | 0.000 |
| Limited work (mental) \| 4 | -0.351 | 0.009 | 0.000 | -0.351 | 0.009 | 0.000 |
| Pain interfered with work \| 1 | 0.101 | 0.009 | 0.000 | 0.101 | 0.009 | 0.000 |
| Pain interfered with work \| 2 | 0.765 | 0.010 | 0.000 | 0.765 | 0.010 | 0.000 |
| Pain interfered with work \| 3 | 1.175 | 0.011 | 0.000 | 1.175 | 0.011 | 0.000 |
| Pain interfered with work \| 4 | 1.780 | 0.016 | 0.000 | 1.780 | 0.016 | 0.000 |
| Felt calm and peaceful \| 1 | -1.031 | 0.011 | 0.000 | -1.031 | 0.011 | 0.000 |
| Felt calm and peaceful \| 2 | 0.318 | 0.009 | 0.000 | 0.318 | 0.009 | 0.000 |
| Felt calm and peaceful \| 3 | 1.109 | 0.011 | 0.000 | 1.109 | 0.011 | 0.000 |
| Felt calm and peaceful \| 4 | 1. 736 | 0.016 | 0.000 | 1. 736 | 0.016 | 0.000 |
| Had a lot of energy \| 1 | -1.097 | 0.011 | 0.000 | -1.097 | 0.011 | 0.000 |
| Had a lot of energy \| 2 | -0.186 | 0.009 | 0.000 | -0.186 | 0.009 | 0.000 |
| Had a lot of energy \| 3 | 0.990 | 0.011 | 0.000 | 0.990 | 0.011 | 0.000 |
| Had a lot of energy \| 4 | 1.619 | 0.015 | 0.000 | 1.619 | 0.015 | 0.000 |
| Felt downhearted and blue \| 1 | -2.033 | 0.020 | 0.000 | -2.033 | 0.020 | 0.000 |
| Felt downhearted and blue \| 2 | -1.431 | 0.013 | 0.000 | -1.431 | 0.013 | 0.000 |
| Felt downhearted and blue \| 3 | -0.569 | 0.009 | 0.000 | -0.569 | 0.009 | 0.000 |
| Felt downhearted and blue \| 4 | 0.225 | 0.009 | 0.000 | 0.225 | 0.009 | 0.000 |
| Problems with social activities \| 1 | -1.870 | 0.017 | 0.000 | -1.870 | 0.017 | 0.000 |
| Problems with social activities \| 2 | -1.375 | 0.013 | 0.000 | -1.375 | 0.013 | 0.000 |
| Problems with social activities \| 3 | -0.784 | 0.010 | 0.000 | -0.784 | 0.010 | 0.000 |
| Problems with social activities \| 4 | -0.305 | 0.009 | 0.000 | -0.305 | 0.009 | 0.000 |
| Item category thresholds (EQ-5D) |  |  |  |  |  |  |
| Mobility \| 1 | 0.842 | 0.010 | 0.000 | 0.842 | 0.010 | 0.000 |
| Mobility \| 2 | 2.561 | 0.034 | 0.000 | 2.561 | 0.034 | 0.000 |
| Self-care \| 1 | 1.580 | 0.014 | 0.000 | 1.580 | 0.014 | 0.000 |
| Self-care \| 2 | 2.396 | 0.028 | 0.000 | 2.396 | 0.028 | 0.000 |
| Usual activities \| 1 | 0.790 | 0.010 | 0.000 | 0.790 | 0.010 | 0.000 |
| Usual activities \| 2 | 1.954 | 0.019 | 0.000 | 1.954 | 0.019 | 0.000 |
| Pain \| 1 | 0.176 | 0.009 | 0.000 | 0.176 | 0.009 | 0.000 |
| Pain \| 2 | 1.673 | 0.015 | 0.000 | 1.673 | 0.015 | 0.000 |
| Anxiety \| 1 | 0.566 | 0.009 | 0.000 | 0.566 | 0.009 | 0.000 |
| Anxiety \| 2 | 1.881 | 0.018 | 0.000 | 1.881 | 0.018 | 0.000 |

## **Table C5:** Sample Demographic Characteristics

|  | **Unidimensional model** | **Bifactor Model** |
| --- | --- | --- |
| **Root mean square error of approximation** | **0.124** | **0.098** |
| **Comparative fit index** | **0.983** | **0.991** |
| **Tucker-Lewis fit** | **0.981** | **0.988** |
| **Standardized Root Mean Square Residual** | **0.102** | **0.073** |

**Face Validity and Predictive Performance**

Figure C3 presents a scatter plot of observed and predicted EQ-5D-3L values from the external dataset (NHMS). These data show similar trends to those in previous studies, especially the underprediction of values for people in full health, as well as those with higher values below full health [25]. The lack of predicted values equivalent to full health can be attributed to the use of the expected utility method. Although this may seem imperfect, it has been shown to produce better predictions when compared to alternative methods such as the Monte Carlo and Most-Likely Probability methods [24].

## **Figure C1:** Scatter Plot of Observed and Predicted EQ-5D-3L Values where the blue dots reflect predictions from the unidimensional LVM and the pink dots reflect predictions from the bifactor LVM. The diagonal line is included to show the point at which predicted and observed EQ-5D-3L would be equivalent.


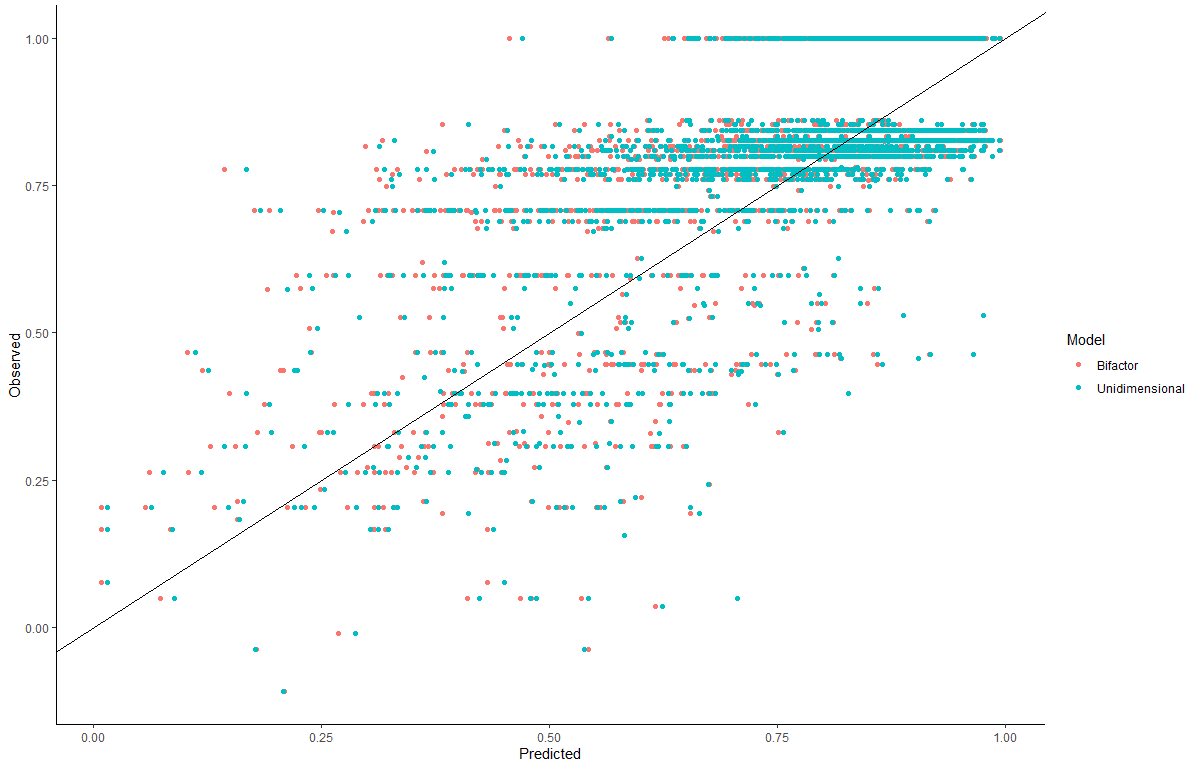


For predicted EQ-5D-3L scores from the unidimensional model in the external dataset, the mean absolute error (MAE) was 0.0958 (95% CI = 0.0957 – 0.0959) and the root mean squared error (RMSE) was 0.1272 (95% CI = 0.1271 – 0.1274). For predicted EQ-5D-3L scores from the bifactor model in the external dataset, the mean absolute error (MAE) was 0.0969 (95% CI = 0.0968 – 0.0970) and the root mean squared error (RMSE) was 0.1290 (95% CI = 0.1288 – 0.1292). The difference in performance between the two models is small, which is due to the same set of parameters (i.e. those related to the general factor) being used to inform predictions. However, the predictions from the bifactor model are theoretically more appropriate than the unidimensional model because it has controlled for instrument-specific variations when estimating parameters in the general factor.

Tables C6 and C7 present these metrics across a range of observed EQ-5D-3L values. No studies were identified in the published literature mapping between the SF-12 Version 2 and the EQ-5D-3L with US population values. The predictive performance of the mapping algorithms developed in this section is, on average, worse than those in the previous section. Despite predictions from this model being worse than those in the model from Appendix B across much of the distribution of EQ-5D-3L values, the predictive performance was better for observed values between 0.5 and 0.7.

## **Table C6:** Mean absolute errors (95% confidence intervals)

| EQ-5D range | Unidimensional model | Bifactor model |
| --- | --- | --- |
| < 0 | 0.3518 (0.3508,0.3527) | 0.3485 (0.3476,0.3494) |
| 0 ≤ x < 0.25 | 0.2431 (0.2426,0.2437) | 0.2391 (0.2386,0.2397) |
| 0.25 ≤ x <0.5 | 0.1609 (0.1604,0.1614) | 0.1558 (0.1553,0.1562) |
| 0.5 ≤ x < 0.7 | 0.1467 (0.1464,0.1471) | 0.1512 (0.1509,0.1515) |
| 0.7 ≤ x < 0.8 | 0.1192 (0.1189,0.1196) | 0.1235 (0.1231,0.1238) |
| 0.8 ≤ x < 0.9 | 0.0842 (0.0841,0.0843) | 0.0854 (0.0853,0.0854) |
| 0.9 ≤ x < 1.0 | 0.0800 (0.0797,0.0802) | 0.0806 (0.0803,0.0808) |

## **Table C7:** Root mean squared errors (95% confidence intervals)

| EQ-5D range | Unidimensional model | Bifactor model |
| --- | --- | --- |
| < 0 | 0.3768 (0.3760,0.3777) | 0.3751 (0.3743,0.3760) |
| 0 ≤ x < 0.25 | 0.2977 (0.2971,0.2982) | 0.2939 (0.2933,0.2944) |
| 0.25 ≤ x <0.5 | 0.1944 (0.1940,0.1948) | 0.1905 (0.1901,0.1909) |
| 0.5 ≤ x < 0.7 | 0.1749 (0.1746,0.1793) | 0.1802 (0.1798,0.1806) |
| 0.7 ≤ x < 0.8 | 0.1602 (0.1597,0.1606) | 0.1656 (0.1651,0.1660) |
| 0.8 ≤ x < 0.9 | 0.1046 (0.1045,0.1047) | 0.1064 (0.1062,0.1065) |
| 0.9 ≤ x < 1.0 | 0.1060 (0.1057,0.1063) | 0.1074 (0.1071,0.1077) |

Figure C4 presents a histogram showing the distribution of prediction errors across a range of values (note the overlap between the distributions for the two methods). Within the external dataset, the percentage of absolute prediction errors below 0.05 was slightly higher for the unidimensional model compared to the bifactor model (36.2% versus 35.9%). The percentage of absolute prediction errors below 0.10 was also slightly higher for the unidimensional model compared to the bifactor model (62.3% versus 61.9%). For reference, values ranging between 0.03 and 0.08 have been purported to represent minimally important differences for the EQ-5D-3L with US population values [31–33].

## **Figure C2:** Histogram of Absolute Prediction Errors

**
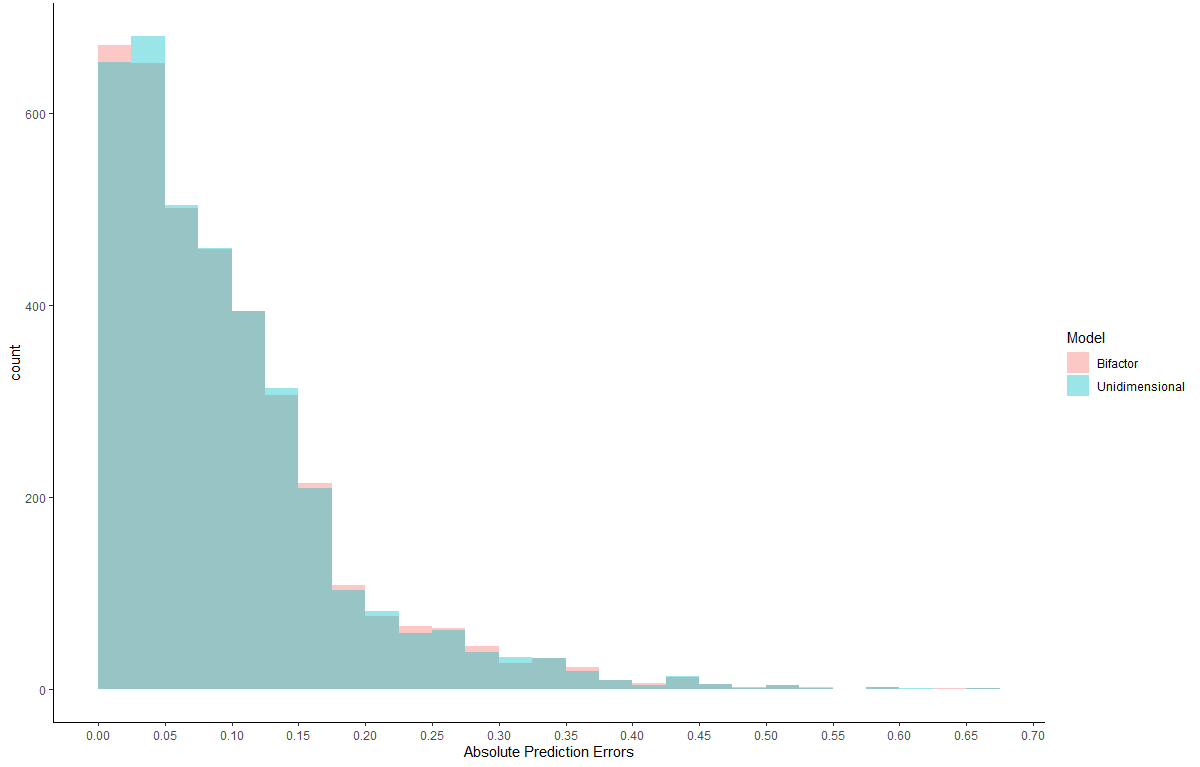
**

**Limitations of the Mapping Algorithms and Further Applications**

The unidimensional mapping algorithm outlined in this section is not without limitations. First, the multiple imputation approach used to handle missing responses to the EQ-5D-3L and SF-12 Version 2 assumed that the data were missing at random. This assumption is only appropriate if the multivariate distributions underlying the observed and missing data are identical [18]. While multiple imputation techniques can also be utilized if the data are missing not at random, this approach requires additional assumptions and presents further challenges in terms of the estimation methods. Another limitation is the misalignment between the sample population used to derive the mapping algorithm (non-condition specific) and the target population for whom the algorithm has been developed to predict EQ-5D-3L responses (people with opioid use disorder). Despite this misalignment, the MEPS data is likely to represent the next best alternative, in lieu of a sample for estimating algorithms in the target population, given that it covers a diverse range of conditions and broad coverage of the health states captured by the two measures. While the primary purpose of the mapping algorithm developed in this section is to predict EQ-5D-3L responses using SF-12 Version 2 responses collected in NIDA trials, it may also prove to be useful in studies beyond the scope of this study. Previous research has indicated that mappings between generic HRQoL instruments are similar across different settings and medical conditions [34]. As such, the mapping algorithm may be useful for predicting EQ-5D-3L responses in US settings and for medical conditions outside the context of this paper. A final point to consider is the methodological question whether the analyzed HRQoL measures are appropriately modelled with models that assume quantitative latent variables. While this has been argued in the past [35, 36], these models assume that all responses in a scale can be reduced to a limited set of causes (one cause in the case of a unidimensional model) that proportionally affect the responses to all items. For instance, if the latent variable in the unidimensional model is increased by one standard deviation, the model predicts a change in all items proportional to their respective loading [37]. Qualitative variation, such as patterns that are mainly characterized by psychological impacts versus those that are characterized by physical impacts, are therefore not considered [38, 39]. While the high loadings in the present study suggest that a substantial amount of inter-individual variation is captured for both measures with either of the latent variable models, further methodological work is needed. An important area for future work would be to explore the consequences of conditions for specific groups or population segments, both empirically as well as in terms of theory building when using such an approach.

**Selection of the Preferred Mapping Algorithm**

The bifactor model was selected as the preferred mapping algorithm for the prediction of EQ-5D values because it met more of the criteria for model fit compared to the unidimensional model. Although the unidimensional model was shown to have slightly better predictive performance than the bifactor model, the difference between the two was, on average, very small such that they are comparable.

**‘Checklist of items to include when reporting a mapping study’** [40]

**Item 1**: Identify the report as a study mapping between outcome measures. State the source measure(s) and generic, preference-based target measure(s) used in the study

See title on page 26.

**Item 2**: Provide a structured abstract including, as applicable: objectives; methods, including data sources and their key characteristics, outcome measures used and estimation and validation strategies; results, including indicators of model performance; conclusions; and implications of key findings

Not applicable.

**Item 3**: Describe the rationale for the mapping study in the context of the broader evidence base

See section under the subheading ‘Study Rationale’ on page 26.

**Item 4**: Specify the research question with reference to the source and target measures used and the disease or population context of the study

See section under the subheading ‘Study Objective’ on page 26.

**Item 5**: Describe how the estimation sample was identified, why it was selected, the methods of recruitment and data collection, and its location(s) or setting(s)

See section under the subheading ‘Estimation Sample’ on page 26.

**Item 6**: If an external validation sample was used, the rationale for selection, the methods of recruitment and data collection, and its location(s) or setting(s) should be described

See section under the subheading ‘External Validation Sample’ on page 26.

**Item 7**: Describe the source and target measures and the methods by which they were applied in the mapping study

See section under the subheading ‘Source and Target Measures’ on page 27.

**Item 8**: Describe the methods used to assess the degree of conceptual overlap between the source and target measures

See section under the subheading ‘Conceptual Overlap Between Measures’ on page 27.

**Item 9**: State how much data were missing and how missing data were handled in the sample(s) used for the analyses

See section under the subheading ‘Exploratory Data Analysis’ on page 27.

**Item 10**: Describe and justify the statistical model(s) used to develop the mapping algorithm

See section under the subheading ‘Model Specification for Mapping Algorithms’ on page 31.

**Item 11**: Describe how predicted scores or utilities are estimated for each model specification

See section under the subheading ‘Prediction of EQ-5D-3L Responses’ on page 31.

**Item 12**: Describe and justify the methods used to validate the mapping algorithm

See section under the subheading ‘Validation of EQ-5D-3L Predictions and Measures of Model Performance’ on page 31.

**Item 13**: State and justify the measure(s) of model performance that determine the choice of the preferred model(s) and describe how these measures were estimated and applied

See section under the subheading ‘Validation of EQ-5D-3L Predictions and Measures of Model Performance’ on page 31.

**Item 14**: State the size of the estimation sample and any validation sample(s) used in the analyses (including both number of individuals and number of observations)

See section under the subheading ‘Sample Description’ on page 31.

**Item 15**: Describe the characteristics of individuals in the sample(s) (or refer back to previous publications giving such information). Provide summary scores for source and target measures, and summarize results of analyses used to assess overlap between the source and target measures

See the section under the subheading ‘Sample Description’ on page 31 and the section under the subheading ‘Conceptual Overlap Between Measures’ on page 27.

**Item 16**: State which model(s) is (are) preferred and justify why this (these) model(s) was (were) chosen

See the section under the subheading ‘Model Outputs’ on page 32.

**Item 17**: Provide all model coefficients and standard errors for the selected model(s). Provide clear guidance on how a user can calculate utility scores based on the outputs of the selected model(s)

See the section under the subheading ‘Model Outputs’ on page 32.

**Item 18**: Report information that enables users to estimate standard errors around mean utility predictions and individual-level variability

See the section under the subheading ‘Model Outputs’ on page 32.

**Item 19**: Present results of model performance, such as measures of prediction accuracy and fit statistics for the selected model(s) in a table or in the text. Provide an assessment of face validity of the selected model(s)

See the section under the subheading ‘Face Validity and Predictive Performance’ on page 35.

**Item 20**: Report details of previously published studies developing mapping algorithms between the same source and target measures and describe differences between the algorithms, in terms of model performance, predictions and coefficients, if applicable

See the section under the subheading ‘Face Validity and Predictive Performance’ on page 35.

**Item 21**: Outline the potential limitations of the mapping algorithm

See the section under the subheading ‘Limitations of the Mapping Algorithms and Further Applications’ on page 36.

**Item 22**: Outline the clinical and research settings in which the mapping algorithm could be used

See the section under the subheading ‘Limitations of the Mapping Algorithms and Further Applications’ on page 36.

**Item 23**: Describe the source(s) of funding and non-monetary support for the study, and the role of the funder(s) in its design, conduct and report. Report any conflicts of interest surrounding the roles of authors and funders

See declarations of interest in the main text.

# APPENDIX D: ADDITIONAL SUMMARY STATISTICS FOR TRIAL DATASETS

In this section, we present a range of graphical displays and descriptive statistics for extra-medical opioid use and treatment with medications for opioid use disorder (MOUD) variables. This information is provided to inform the inclusion of interaction terms for specific combinations of predictors in the regression analysis. We sought to include interactions between the main fixed effects variables corresponding to opioid use (either medicated or non-medicated) to account for cases where participants reported using multiple types of opioids. Underlying this approach is an explicit assumption that the statistical association between each individual predictors and the dependent variable (EQ-5D-3L index values) depends on the value/level of the other predictors. This assumption is considered appropriate given that the different opioid types represent alternatives within the same drug class and, as such, cannot be considered independently of each other. Before we attempt to include these interaction terms in the regression, we must check that there is sufficient overlap across the distribution of observed predictor values to ensure that their estimation is feasible.

Figures D1 to D3 contain plots showing the distribution of self-reported days of extra-medical opioid use for each of the three drug types (heroin, other opiate, and methadone) stratified according to the responses associated with the other drug types. The covariate patterns in each of these plots indicates that there is sufficient overlap between each of the variables to estimate parameters associated with interaction terms. Tables D1 to D3 and Figures D4 to D6 explore the distribution of self-reported days of extra-medical opioid use for each of the three drug types, conditional on whether study participants are receiving MOUD treatment at the same time. The covariate patterns for self-reported heroin use and other opiates use indicate that there is sufficient coverage across the MOUD treatment responses to justify including the corresponding interaction terms. However, due to the small numbers of MOUD-treated participants reporting any methadone use, an interaction between these variables was not included in the model.

## **Figure D1:** Histograms displaying responses to the self-reported number of days of heroin use conditional on the self-reported number of days using other opiates


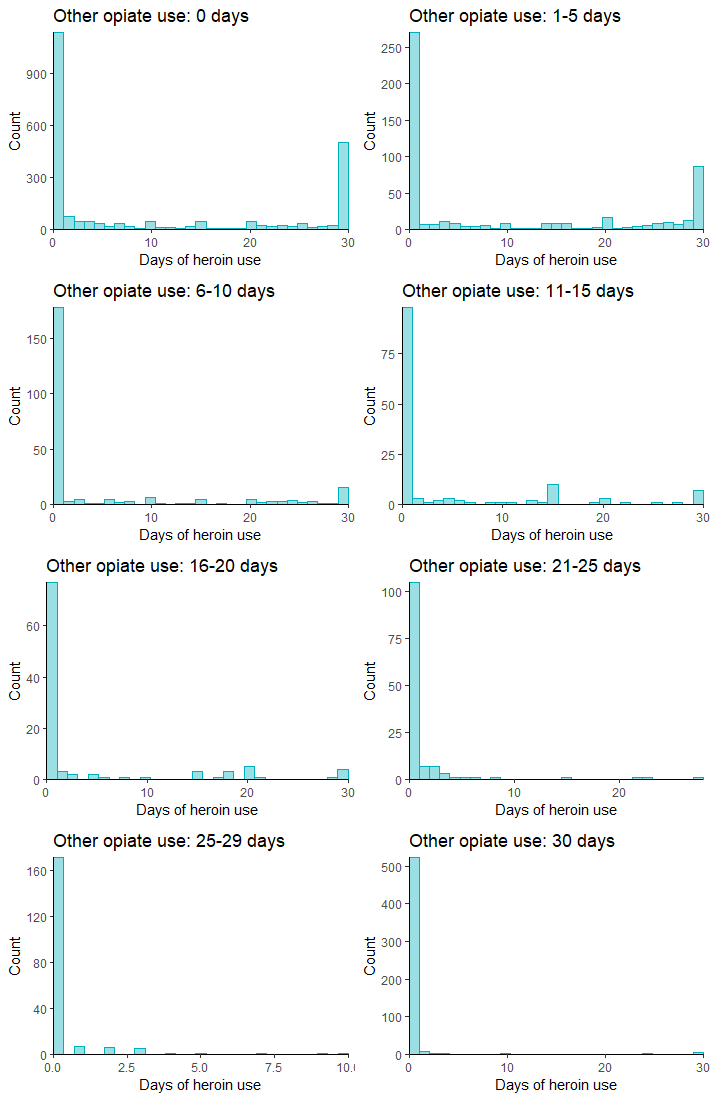


## **Figure D2:** Histograms displaying responses to the self-reported number of days of heroin use conditional on the self-reported number of days using methadone


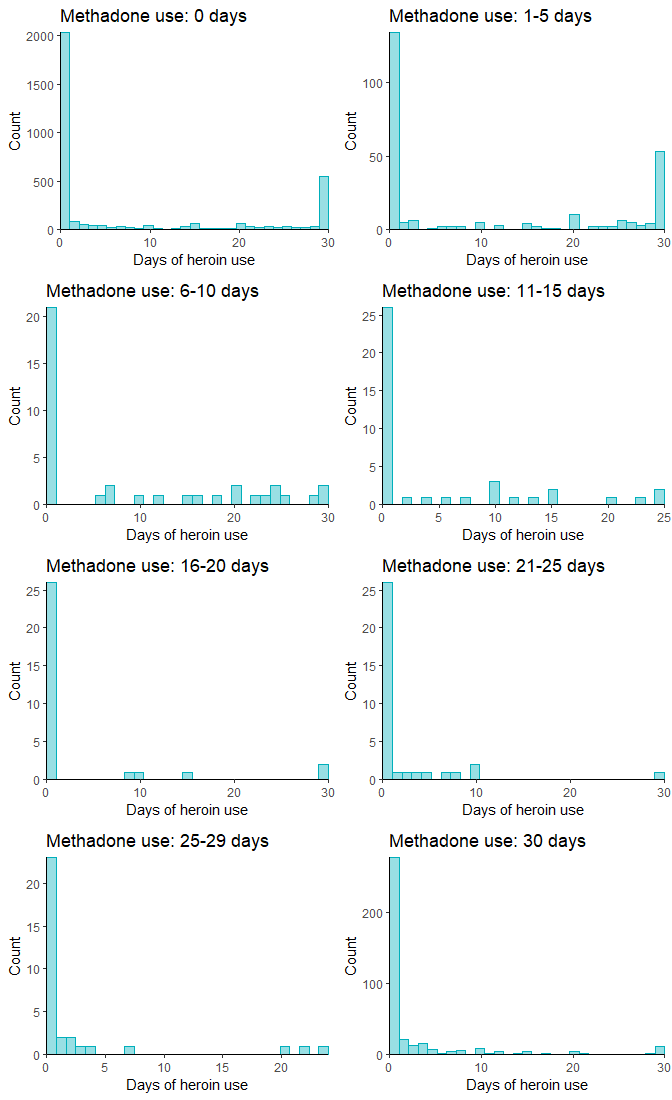


## **Figure D3:** Histograms displaying responses to the self-reported number of days of other opiate use conditional on the self-reported number of days using methadone


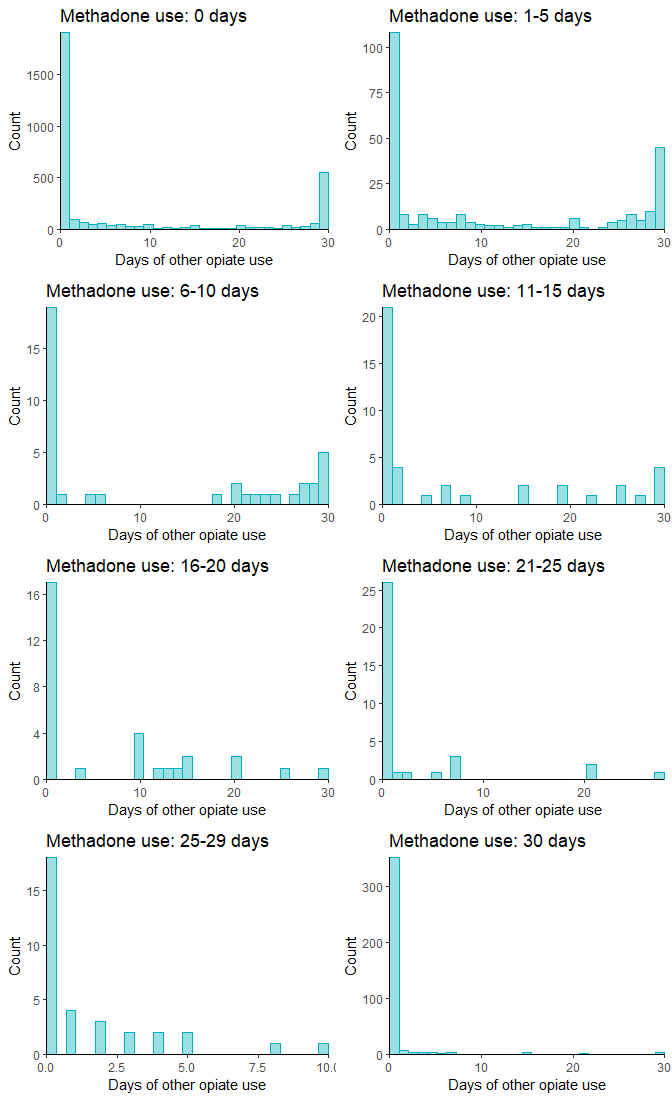


## **Table D1:** Frequency distribution displaying responses to the self-reported number of days of heroin use and treatment status with medications for opioid use disorder (MOUD)

|  |  | Number of days of heroin use | |
| --- | --- | --- | --- |
|  |  | None | One or more |
| Treatment with MOUD | No | 2,347 (56%) | 1,600 (38%) |
|  | Yes | 101 (2%) | 142 (3%) |

## **Figure D4:** Histogram displaying responses to the self-reported number of days of heroin use conditional on treatment status with medications for opioid use disorder (MOUD)


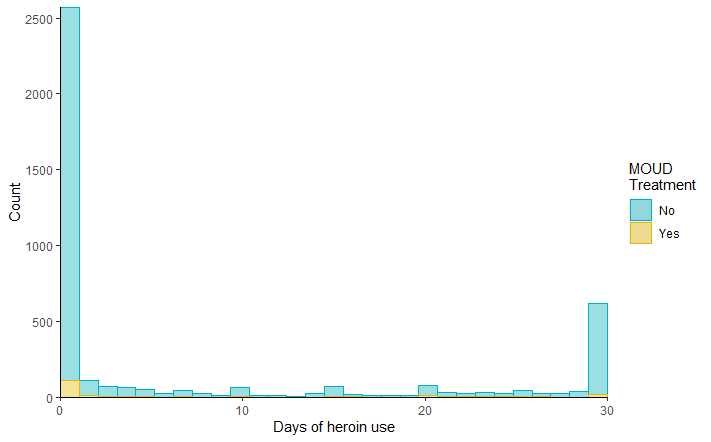


## **Table D2:** Frequency distribution displaying responses to the self-reported number of days of other opiate use and treatment status with medications for opioid use disorder (MOUD)

|  |  | Number of days of other opiate use | |
| --- | --- | --- | --- |
|  |  | None | One or more |
| Treatment with MOUD | No | 2,130 (51%) | 1,817 (43%) |
|  | Yes | 168 (4%) | 75 (2%) |

## **Figure D5:** Histogram displaying responses to the self-reported number of days of other opiate use conditional on treatment status with medications for opioid use disorder (MOUD)


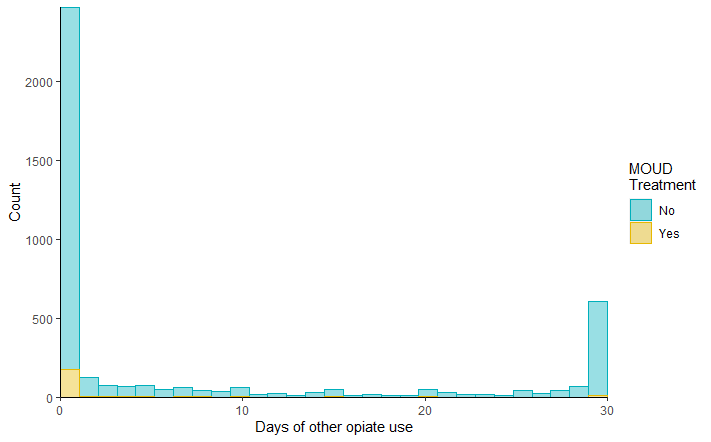


## **Table D3:** Frequency distribution displaying responses to the self-reported number of days of methadone use and treatment status with medications for opioid use disorder (MOUD)

|  |  | Number of days of methadone use | |
| --- | --- | --- | --- |
|  |  | None | One or more |
| Treatment with MOUD | No | 3,144 (75%) | 803 (19%) |
|  | Yes | 226 (5%) | 17 (<1%) |

## **Figure D6:** Histogram displaying responses to the self-reported number of days of methadone use conditional on treatment status with medications for opioid use disorder (MOUD)


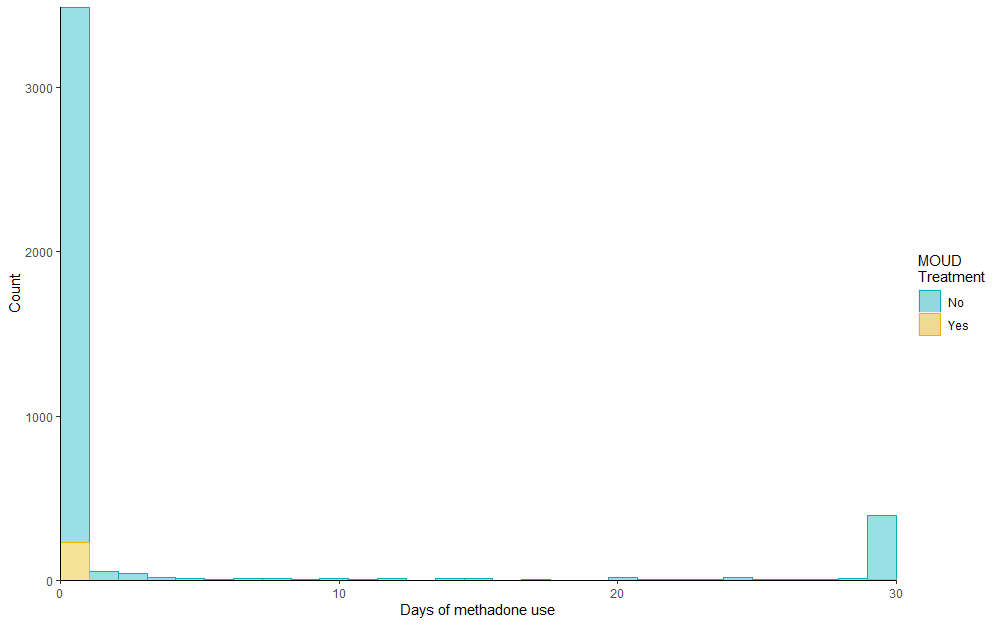


# APPENDIX E: RESULTS FROM REGRESSION ANALYSES

## **Table E1:** Results from the main analysis on the beta-binomial scale

| **Model parameter number** | **Model parameter description** | **Estimate** | **Standard error** | **P-value** |
| --- | --- | --- | --- | --- |
| 1 | Intercept | 2.42 | 0.18 | <0.001 |
| 2 | Days of heroin use in past 30 days | -0.01 | 0.01 | 0.09 |
| 3 | Days of other opiate use in past 30 days | -0.01 | 0.004 | 0.002 |
| 4 | Days of methadone use in past 30 days | -0.01 | 0.01 | 0.12 |
| 5 | Treatment with MOUD | 0.06 | 0.29 | 0.84 |
| 6 | Injecting as the main route of administration | -0.29 | 0.12 | 0.02 |
| 7 | HIV positive diagnosis | -0.46 | 0.19 | 0.02 |
| 8 | Age (years) | -0.01 | 0.004 | 0.03 |
| 9 | Interaction between variables 2 and 3 | -0.001 | 0.001 | 0.31 |
| 10 | Interaction between variables 2 and 4 | <0.001 | 0.001 | 0.72 |
| 11 | Interaction between variables 3 and 4 | -0.001 | 0.001 | 0.41 |
| 12 | Interaction between variables 2 and 5 | -0.01 | 0.02 | 0.63 |
| 13 | Interaction between variables 3 and 5 | -0.01 | 0.02 | 0.75 |

MOUD = medications for opioid use disorder; HIV = human immunodeficiency virus.

Number of observations in the analysis = 4,190

Number of study participants in the analysis = 1,777

## **Table E2:** Results from the secondary analyses on the beta-binomial scale

| **Model parameter number** | **Model parameter description** | **Estimate** | **Standard error** | **P-value** |
| --- | --- | --- | --- | --- |
| 1 | Intercept | 2.44 | 0.31 | <0.001 |
| 2 | Days of heroin use in past 30 days | -0.004 | 0.01 | 0.61 |
| 3 | Days of other opiate use in past 30 days | -0.01 | 0.01 | 0.37 |
| 4 | Days of methadone use in past 30 days | -0.06 | 0.12 | 0.63 |
| 5 | Treatment with MOUD | 0.46 | 0.53 | 0.39 |
| 6 | Injecting as the main route of administration | -0.10 | 0.19 | 0.61 |
| 7 | SOWS measure | -0.04 | 0.01 | <0.001 |
| 8 | Age (years) | -0.004 | 0.01 | 0.66 |
| 9 | Interaction between variables 2 and 3 | >-0.001 | <0.001 | 0.66 |
| 10 | Interaction between variables 2 and 4 | 0.003 | 0.005 | 0.56 |
| 11 | Interaction between variables 3 and 4 | 0.002 | 0.006 | 0.71 |
| 12 | Interaction between variables 2 and 5 | -0.02 | 0.02 | 0.49 |
| 13 | Interaction between variables 3 and 5 | -0.03 | 0.03 | 0.40 |

MOUD = medications for opioid use disorder; SOWS = Subjective Opiate Withdrawal Scale.

Number of observations in the analysis = 1,382

Number of study participants in the analysis = 544

# APPENDIX F: RESULTS FROM SENSITIVITY ANALYSIS

We sought to account for variability in health state utilities across the six clinical trials as a sensitivity analysis. Initially, a three-level hierarchical model structure was tested which accounted for correlations between repeated observations (for individual study participants) in addition to correlations between study participants in the same clinical trial (the former being nested within the latter). This approach was ultimately not supported by the data. Even for a parsimonious model specification without independent variables (i.e. intercepts only), the analysis failed to converge. Evidence from the published literature suggests that this situation is not uncommon when fitting mixed-effects models [45, 46]. As an alternative, we explored the inclusion of a fixed-effects independent variable capturing the study that patients were enrolled in. To perform this analysis, we dropped data from study NCT01612169 because of concerns about multicollinearity between the study variable and the HIV variable, given that almost all patients living with HIV were in this study. A Wald test was performed to confirm whether the variable “data” was statistically significant for the models which revealed a p-value of 0.11. This suggests that study-level effects did not have a significant impact on our findings. Table F1 shows results from the regression analysis, with outputs on the beta-binomial scale and study coefficients are measured relative to study NCT00032955. Table F2 shows the average marginal effects associated with the contrasts from this analysis. The inclusion study-specific dummy variables did not yield any changes the mean predicted HSU values (shown in Table F3) larger than 0.031.

## **Table F1:** Results from the regression sensitivity analysis on the beta-binomial scale

| **Model parameter number** | **Model parameter description** | **Estimate** | **Standard error** | **P-value** |
| --- | --- | --- | --- | --- |
| 1 | Intercept | 2.70 | 0.28 | <0.001 |
| 2 | Days of heroin use in past 30 days | -0.01 | 0.01 | 0.04 |
| 3 | Days of other opiate use in past 30 days | -0.02 | 0.01 | 0.005 |
| 4 | Days of methadone use in past 30 days | 0.002 | 0.01 | 0.84 |
| 5 | Treatment with MOUD | 0.44 | 0.43 | 0.32 |
| 6 | Injecting as the main route of administration | -0.29 | 0.13 | 0.03 |
| 7 | Age (years) | -0.01 | 0.004 | 0.03 |
| 8 | Interaction between variables 2 and 3 | -0.001 | 0.001 | 0.41 |
| 9 | Interaction between variables 2 and 4 | <0.001 | 0.001 | 0.67 |
| 10 | Interaction between variables 3 and 4 | -0.001 | 0.001 | 0.32 |
| 11 | Interaction between variables 2 and 5 | -0.018 | 0.02 | 0.39 |
| 12 | Interaction between variables 3 and 5 | -0.020 | 0.03 | 0.44 |
| 13 | Study NCT00032968 dummy variable | -0.06 | 0.24 | 0.81 |
| 14 | Study NCT00067158 dummy variable | -0.66 | 0.33 | 0.04 |
| 15 | Study NCT00316277 dummy variable | -0.21 | 0.24 | 0.37 |
| 16 | Study NCT02032433 dummy variable | -0.32 | 0.21 | 0.13 |

MOUD = medications for opioid use disorder.

Number of observations in the analysis = 3,976

Number of study participants in the analysis = 1,691

## **Table F2:** Estimated Health State Utilities’ (HSU) effects associated with contrasts for the predictor variables from the regression sensitivity analysis

| Variable | Contrast | Estimated difference in HSU (mean) | Lower 95% Confidence Interval | Upper 95% Confidence Interval |
| --- | --- | --- | --- | --- |
| Days of heroin use | +1 day | -0.0023 | -0.0371 | 0.0325 |
| Days of other opiate use | +1 day | -0.0035 | -0.0385 | 0.0315 |
| Days of methadone use | +1 day | -0.0004 | -0.0436 | 0.0428 |
| MOUD | Yes vs. no | -0.0013 | -0.0486 | 0.0742 |
| Injecting as the main route of administration | Yes vs. no | -0.0422 | -0.0765 | -0.0079 |
| Age (years) | +1 year | -0.0014 | -0.0058 | 0.0030 |
| Study NCT00032955 dummy variable | NCT00032968 vs. NCT00032955 | -0.0066 | -0.0595 | 0.0463 |
| Study NCT00032968 dummy variable | NCT00067158 vs. NCT00032955 | -0.0958 | -0.2150 | 0.0234 |
| Study NCT00067158 dummy variable | NCT00316277 vs. NCT00032955 | -0.0267 | -0.0866 | 0.0332 |
| Study NCT02032433 dummy variable | NCT02032433 vs. NCT00032955 | -0.0407 | -0.0943 | 0.0130 |

MOUD = medications for opioid use disorders.

## **Table F3:** Predicted Health State Utility (HSU) values for health states of interest in opioid use disorders (OUD) models

| Health state number | Days of heroin use | Days of other opiate use | Injecting as the main route of administration | Receipt of MOUD treatment | Mean HSU estimate | Lower 95% confidence interval | Upper 95% confidence interval |
| --- | --- | --- | --- | --- | --- | --- | --- |
| 1 | 30 | 0 | Yes | No | 0.775 | 0.728 | 0.815 |
| 2 | 0 | 30 | No | No | 0.802 | 0.740 | 0.851 |
| 3 | 15 | 0 | Yes | Yes | 0.831 | 0.749 | 0.890 |
| 4 | 0 | 15 | No | Yes | 0.857 | 0.747 | 0.923 |
| 5 | 0 | 0 | No | No | 0.870 | 0.844 | 0.891 |
| 6 | 0 | 0 | No | Yes | 0.912 | 0.816 | 0.960 |

MOUD = medications for opioid use disorders.

# References

1. Lee, J. D., Nunes Jr, E. V., Novo, P., Bachrach, K., Bailey, G. L., Bhatt, S., … others. (2018). Comparative effectiveness of extended-release naltrexone versus buprenorphine-naloxone for opioid relapse prevention (X: BOT): a multicentre, open-label, randomised controlled trial. *The Lancet*, *391*(10118), 309–318.

2. Ling, W., Amass, L., Shoptaw, S., Annon, J. J., Hillhouse, M., Babcock, D., … others. (2005). A multi-center randomized trial of buprenorphine–naloxone versus clonidine for opioid, detoxification: findings from the National Institute on Drug Abuse Clinical Trials Network. *Addiction*, *100*(8), 1090–1100.

3. Metsch, L. R., Feaster, D. J., Gooden, L., Matheson, T., Stitzer, M., Das, M., … others. (2016). Effect of patient navigation with or without financial incentives on viral suppression among hospitalized patients with HIV infection and substance use: a randomized clinical trial. *Jama*, *316*(2), 156–170.

4. Reid, M. S., Fallon, B., Sonne, S., Flammino, F., Nunes, E. V., Jiang, H., … others. (2008). Smoking cessation treatment in community-based substance abuse rehabilitation programs. *Journal of substance abuse treatment*, *35*(1), 68–77.

5. Weiss, R. D., Potter, J. S., Fiellin, D. A., Byrne, M., Connery, H. S., Dickinson, W., … others. (2011). Adjunctive counseling during brief and extended buprenorphine-naloxone treatment for prescription opioid dependence: a 2-phase randomized controlled trial. *Archives of general psychiatry*, *68*(12), 1238–1246.

6. Sullivan, P. W., & Ghushchyan, V. (2006). Preference-based EQ-5D index scores for chronic conditions in the United States. *Medical Decision Making*, *26*(4), 410–420.

7. Shaw, J. W., Johnson, J. A., & Coons, S. J. (2005). US valuation of the EQ-5D health states: development and testing of the D1 valuation model. *Medical care*, 203–220.

8. Gray, A. M., Rivero-Arias, O., & Clarke, P. M. (2006). Estimating the association between SF-12 responses and EQ-5D utility values by response mapping. *Medical Decision Making*, *26*(1), 18–29.

9. Sullivan, P. W., & Ghushchyan, V. (2006). Mapping the EQ-5D index from the SF-12: US general population preferences in a nationally representative sample. *Medical Decision Making*, *26*(4), 401–409.

10. Chowdhury, S. R., Machlin, S. R., & Gwet, K. L. (2019). Methodology report# 33: sample designs of the medical expenditure panel survey household component, 1996–2006 and 2007–2016. *Rockville, MD, Agency for Healthcare Research and Quality*.

11. Botman, S. L., Moore, T. F., Moriarity, C. L., & Parsons, V. L. (2000). *Design and estimation for the national health interview survey, 1995-2004*. National Center for Health Statistics.

12. Nosyk, B., Sun, H., Guh, D. P., Oviedo-Joekes, E., Marsh, D. C., Brissette, S., … Anis, A. H. (2010). The quality of eight health status measures were compared for chronic opioid dependence. *Journal of clinical epidemiology*, *63*(10), 1132–1144.

13. EuroQol Research Foundation. (2018). *EQ-5D-3L User Guide*. Retrieved from https://euroqol.org/publications/user-guides

14. Ware Jr, J., Kosinski, M., & Keller, S. (1998). SF-12 (R): How to score the SF-12 (R) Physical and Mental Health Summary Scale. Boston: Quality Metric. Inc.

15. Johnson, J. A., & Coons, S. J. (1998). Comparison of the EQ-5D and SF-12 in an adult US sample. *Quality of Life Research*, *7*(2), 155–166.

16. Johnson, J. A., & Pickard, A. S. (2000). Comparison of the EQ-5D and SF-12 health surveys in a general population survey in Alberta, Canada. *Medical care*, *38*(1), 115–121.

17. Little, R. J. (1988). A test of missing completely at random for multivariate data with missing values. *Journal of the American statistical Association*, *83*(404), 1198–1202.

18. Van Buuren, S., & Groothuis-Oudshoorn, K. (2011). mice: Multivariate imputation by chained equations in R. *Journal of statistical software*, *45*, 1–67.

19. Lu, G., Kounali, D., & Ades, A. (2014). Simultaneous multioutcome synthesis and mapping of treatment effects to a common scale. *Value in Health*, *17*(2), 280–287.

20. Edwards, M. C., Wirth, R., Houts, C. R., & Xi, N. (2012). Categorical data in the structural equation modeling framework.

21. Rosseel, Y. (2012). lavaan: An R package for structural equation modeling. *Journal of statistical software*, *48*, 1–36.

22. Reise, S. P. (2012). The rediscovery of bifactor measurement models. *Multivariate behavioral research*, *47*(5), 667–696.

23. Gibbons, R. D., Perraillon, M. C., & Kim, J. B. (2014). Item response theory approaches to harmonization and research synthesis. *Health Services and Outcomes Research Methodology*, *14*(4), 213–231.

24. Le, Q. A., & Doctor, J. N. (2011). Probabilistic mapping of descriptive health status responses onto health state utilities using Bayesian networks: an empirical analysis converting SF-12 into EQ-5D utility index in a national US sample. *Medical care*, 451–460.

25. Longworth, L., & Rowen, D. (2013). Mapping to obtain EQ-5D utility values for use in NICE health technology assessments. *Value in health*, *16*(1), 202–210.

26. Brazier, J. E., Yang, Y., Tsuchiya, A., & Rowen, D. L. (2010). A review of studies mapping (or cross walking) non-preference based measures of health to generic preference-based measures. *The European journal of health economics*, *11*(2), 215–225.

27. Kline, R. B. (2015). *Principles and practice of structural equation modeling*. Guilford publications.

28. Boateng, G. O., Neilands, T. B., Frongillo, E. A., Melgar-Quiñonez, H. R., & Young, S. L. (2018). Best practices for developing and validating scales for health, social, and behavioral research: a primer. *Frontiers in public health*, *6*, 149.

29. Coca Perraillon, M., Shih, Y.-C. T., & Thisted, R. A. (2015). Predicting the EQ-5D-3L preference index from the SF-12 health survey in a national US sample: a finite mixture approach. *Medical Decision Making*, *35*(7), 888–901.

30. Franks, P., Lubetkin, E. I., Gold, M. R., & Tancredi, D. J. (2003). Mapping the SF-12 to preference-based instruments: convergent validity in a low-income, minority population. *Medical Care*, 1277–1283.

31. Kohn, C. G., Sidovar, M. F., Kaur, K., Zhu, Y., & Coleman, C. I. (2014). Estimating a minimal clinically important difference for the EuroQol 5-Dimension health status index in persons with multiple sclerosis. *Health and Quality of Life Outcomes*, *12*(1), 1–5.

32. Pickard, A. S., Neary, M. P., & Cella, D. (2007). Estimation of minimally important differences in EQ-5D utility and VAS scores in cancer. *Health and quality of life outcomes*, *5*(1), 1–8.

33. Sullivan, P. W., Lawrence, W. F., & Ghushchyan, V. (2005). A national catalog of preference-based scores for chronic conditions in the United States. *Medical care*, 736–749.

34. Rowen, D., Brazier, J., & Roberts, J. (2009). Mapping SF-36 onto the EQ-5D index: how reliable is the relationship? *Health and quality of life outcomes*, *7*(1), 1–9.

35. Gibbons, R. D., Perraillon, M. C., & Kim, J. B. (2014). Item response theory approaches to harmonization and research synthesis. *Health Services and Outcomes Research Methodology*, *14*(4), 213–231.

36. Lu, G., Kounali, D., & Ades, A. (2014). Simultaneous multioutcome synthesis and mapping of treatment effects to a common scale. *Value in Health*, *17*(2), 280–287.

37. Costa, D. S. (2015). Reflective, causal, and composite indicators of quality of life: A conceptual or an empirical distinction? *Quality of Life Research*, *24*, 2057–2065.

38. Cohen, P., Cohen, J., Teresi, J., Marchi, M., & Velez, C. N. (1990). Problems in the measurement of latent variables in structural equations causal models. *Applied Psychological Measurement*, *14*(2), 183–196.

39. Feng, Y.-S., Jiang, R., Pickard, A. S., & Kohlmann, T. (2021). Combining EQ-5D-5L items into a level summary score: demonstrating feasibility using non-parametric item response theory using an international dataset. *Quality of Life Research*, 1–13.

40. Petrou, S., Rivero-Arias, O., Dakin, H., Longworth, L., Oppe, M., Froud, R., & Gray, A. (2015). The MAPS reporting statement for studies mapping onto generic preference-based outcome measures: explanation and elaboration. *Pharmacoeconomics*, *33*(10), 993–1011.

41. Fryback, D. G., Dunham, N. C., Palta, M., Hanmer, J., Buechner, J., Cherepanov, D., … others. (2007). US norms for six generic health-related quality-of-life indexes from the National Health Measurement study. *Medical care*, *45*(12), 1162.

42. Ware Jr, J., Kosinski, M., Turner-Bowker, D., & Gandek, B. (2002). User’s Manual for the SF-12v2® Health Survey With a Supplement Documenting SF-12® Health Survey) Lincoln. *RI: QualityMetric Incorporated*, *2009*.

43. Cheak-Zamora, N. C., Wyrwich, K. W., & McBride, T. D. (2009). Reliability and validity of the SF-12v2 in the medical expenditure panel survey. *Quality of Life Research*, *18*(6), 727–735.

44. Fleishman, J. A., Selim, A. J., & Kazis, L. E. (2010). Deriving SF-12v2 physical and mental health summary scores: a comparison of different scoring algorithms. *Quality of Life Research*, *19*(2), 231–241.

45. Bates, D., Kliegl, R., Vasishth, S., & Baayen, H. (2015). Parsimonious mixed models. *arXiv preprint arXiv:1506.04967*.

46. Matuschek, H., Kliegl, R., Vasishth, S., Baayen, H., & Bates, D. (2017). Balancing Type I error and power in linear mixed models. *Journal of memory and language*, *94*, 305–315.
